# Revealing nutritional requirements of MICP-relevant *Sporosarcina pasteurii* DSM33 for growth improvement in chemically defined and complex media

**DOI:** 10.1038/s41598-020-79904-9

**Published:** 2020-12-31

**Authors:** Frédéric M. Lapierre, Jakob Schmid, Benjamin Ederer, Nina Ihling, Jochen Büchs, Robert Huber

**Affiliations:** 1 Munich University of Applied Sciences, 80335 Munich, Germany; 2 Chair of Biochemical Engineering (AVT.BioVT), RWTH Aachen University, 52074 Aachen, Germany

**Keywords:** Biomineralization, Applied microbiology

## Abstract

Microbial induced calcite precipitation (MICP) based on ureolysis has a high potential for many applications, e.g. restoration of construction materials. The gram-positive bacterium *Sporosarcina pasteurii* is the most commonly used microorganism for MICP due to its high ureolytic activity. However, *Sporosarcina pasteurii* is so far cultivated almost exclusively in complex media, which only results in moderate biomass concentrations at the best. Cultivation of *Sporosarcina pasteurii* must be strongly improved in order to make technological application of MICP economically feasible. The growth of *Sporosarcina pasteurii* DSM 33 was boosted by detecting auxotrophic deficiencies (L-methionine, L-cysteine, thiamine, nicotinic acid), nutritional requirements (phosphate, trace elements) and useful carbon sources (glucose, maltose, lactose, fructose, sucrose, acetate, L-proline, L-alanine). These were determined by microplate cultivations with online monitoring of biomass in a chemically defined medium and systematically omitting or substituting medium components. Persisting growth limitations were also detected, allowing further improvement of the chemically defined medium by the addition of glutamate group amino acids. Common complex media based on peptone and yeast extract were supplemented based on these findings. Optical density at the end of each cultivation of the improved peptone and yeast extract media roughly increased fivefold respectively. A maximum OD600 of 26.6 ± 0.7 (CDW: 17.1 ± 0.5 g/L) was reached with the improved yeast extract medium. Finally, culture performance and media improvement was analysed by measuring the oxygen transfer rate as well as the backscatter during shake flask cultivation.

## Introduction

Microbial induced calcite precipitation (MICP) is considered for several applications, such as soil reinforcement and restoration of construction materials like limestone or concrete^1,2^, metal and radionuclide remediation^3–5^ and CO$_2$ sequestration^6^. Precipitation based on the presence of carbonate can be caused by a multitude of metabolic pathways like photosynthesis, denitrification or sulphate reduction^7,8^. However, precipitation induced by ureolysis is especially promising as carbonate is produced directly alongside with ammonium ions by hydrolysis of urea^9^. The overall process results in an increase of the environmental pH value and thus in ideal conditions for calcium carbonate (calcite) precipitation in the presence of calcium ions.

The gram-positive bacterium *Sporosarcina pasteurii*, formerly referred to as *Bacillus pasteurii*, is known to have one of the highest urease activities compared to other organisms^10^, while being not pathogenic. Its ability to form endospores as well as its alkaliphilic and halophilic properties^11^ make it suitable for application on construction materials like concrete. Therefore, *S. pasteurii* is the most used microorganism for MICP^1,12^ and consequently gets a lot of attention in recent studies with the goal of increasing urease activity or growth^13–15^.

*S. pasteurii* is cultivated almost exclusively in complex media, typically containing yeast extract or peptone^10,12,14,16,17^, as these are convenient to use and well established. To the best of our knowledge, only Kuhlmann and Bremer^18^ successfully used a chemically defined medium in order to study ectoine biosynthesis. Growth in their chemically defined medium was similar to growth observed in complex media^18^. Additionally, some cultivation experiments are using waste water from manifold sources as cultivation medium^17,19^. This approach is expected to be profitable, as waste water can be seen as a very cheap or even free resource for industrial scale cultivation. However, although numerous cultivation media for *S. pasteurii* are published so far, they only lead to moderate or even low bacterial growth. Typically, at the end of the cultivation, only an optical density (OD) below 5 at a wavelength of 600 nm is reached . Up to this point, no cultivation protocol leading to notably higher cell densities has been published. Such a cultivation protocol is urgently needed, as improving *S. pasteurii* cultivation performance is a prerequisite to make MICP economically more feasible for industrial applications^1,20–22^. Therefore, a cheap medium for efficient cultivation based on the nutritional requirements of *S. pasteurii* needs to be developed.

Using complex media makes it very difficult or even impossible to exactly determine growth limiting substrates or growth inhibiting components, as the precise composition of the medium is tedious to determine^23^. Components of complex media are also known to vary in their nutrient composition, having an impact on bacterial growth rate, product yield and product quality^24^. This complicates reproducible cultivation and is therefore hindering systematic process optimization^25,26^.

Contrary to complex media, growth limitations due to auxotrophic deficiencies and nutritional requirements of an organism can be identified very easily when using chemically defined media^25–27^. Knowledge about nutritional requirements and auxotrophic deficiencies can be applied to improve cultivation performance^23^. The International Union of Pure and Applied Chemistry defines the term auxotrophy as the inability of an organism to synthesize a particular organic component required for its growth^28^. Also, all microorganisms require inorganic nutrients (e.g. phosphorus, potassium, sulphur) as they cannot be synthesized by a microorganism itself and therefore have to be present in the culture medium. Using chemically defined media additionally allows gathering basic information about bacterial metabolism, e.g. which carbohydrates are metabolised^26^.

The auxotrophic deficiencies and nutritional requirements of *S. pasteurii* strains are described to be some amino acids, which are not further defined, thiamine, biotin, nicotinic acid, ammonia^29^ and glutamine^30^. However, variations of these deficiencies are described from strain to strain^29^.

Here, we further examine the nutritional requirements and auxotrophic deficiencies of *S. pasteurii* DSM33 by cultivation in chemically defined media using a high-throughput online-monitoring microbioreactor system based on microplate cultivation (BioLector). This allows for fast screening of bacterial nutrient demands, as numerous batch cultivation experiments can be carried out simultaneously^31^. After this, an improved chemically defined medium is used in another microplate cultivation experiment to define useful carbon sources for the microorganism by substituting glucose as the main carbon source with other common substrates. Results are then applied to supplement cheap complex media in order to further improve biomass concentration and create a cheap but efficient culture medium. Finally, the oxygen transfer rate (OTR) and bacterial growth during shake-flask cultivation are determined with online-monitoring tools (KuhnerTOM, CellGrowthQuantifier) in order to analyse culture performance and media improvement. An overview of the cultivation experiments and methods is given in Supplementary Figure S1 online.

## Results and discussion

### Examination of nutritional requirements of *S. pasteurii*

In order to define auxotrophic deficiencies and nutritional requirements, *S. pasteurii* DSM33 was cultivated in variations of a chemically defined medium. Components were omitted from the medium to check if they are required for growth. If no growth was detected, the component omitted from the medium was considered as crucial for *S. pasteurii*. The same was assumed, when strongly limited growth was observed. Here we define strongly limited growth as a final backscatter less than 40 % compared to growth in unmodified medium (corresponding to Table 1), similar to Aller et al.^23^.

**Figure 1:**
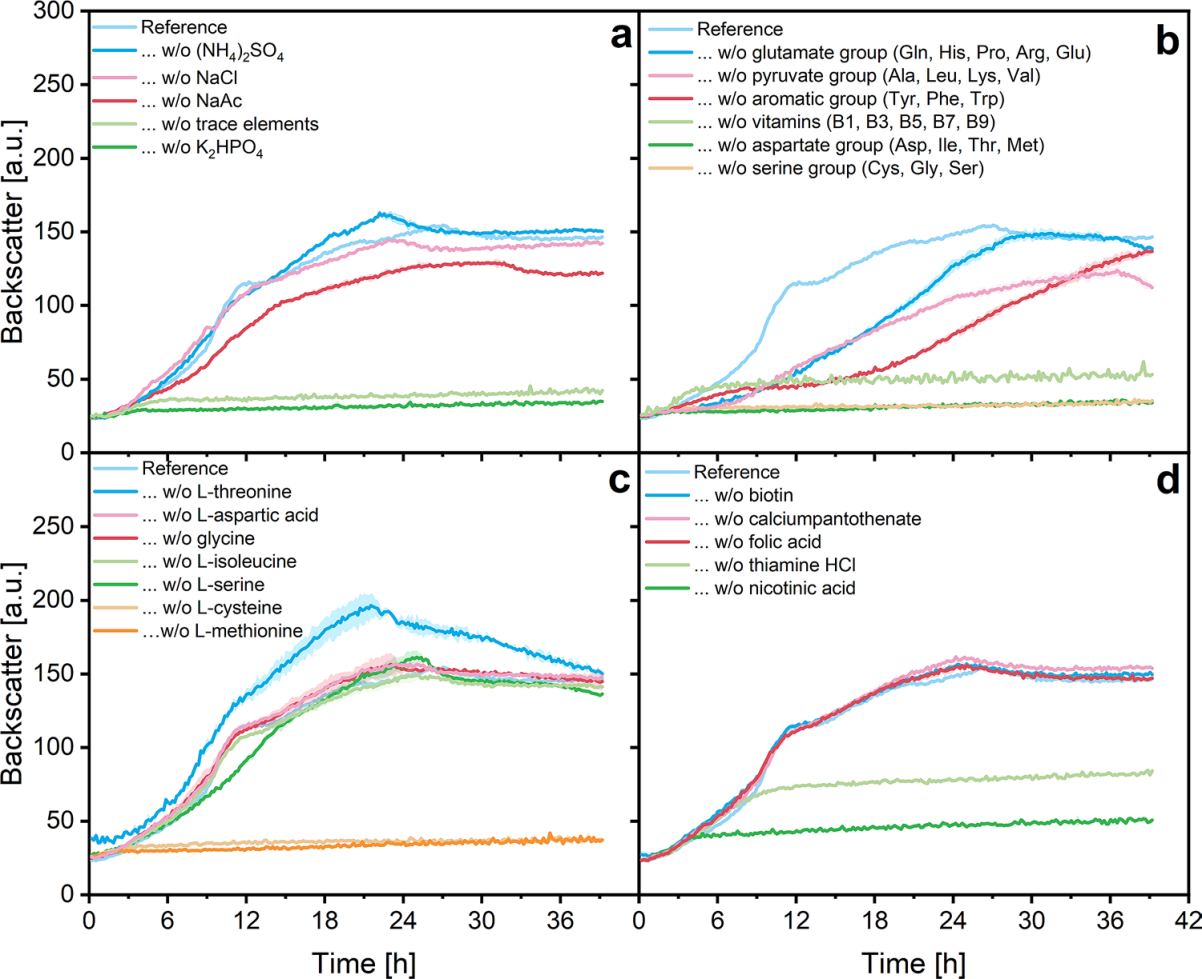
Backscatter data from a microplate cultivation of *S. pasteurii* in modified chemically defined media to determine nutritional requirements and auxotrophic deficiencies. Illustrated are backscatter data from different growth curves after leaving out grouped or single components from the chemically defined medium (Table 1). Culture conditions: 48-well Flower Well Plate, filling volume 800 $\upmu$L, shaking frequency 1200 rpm, shaking diameter 3 mm and temperature 30 $^\circ$C. Here, arithmetic mean values derived from biological replicates (N = 2, N = 1 for w/o vitamins) are shown. The error bands depict the standard deviation.

During a first experimental run, components were omitted from the medium in groups to reduce the overall number of experiments. When no or strongly limited growth was detected in the first run, all components of a group were omitted one by one in a second experiment to determine, which of the groups components were essential for growth. Finally, all cultivation experiments were again carried out as duplicates in one single microplate to confirm previous observations. Results from this final run are illustrated in Fig. 1. As a reference, the microorganism was cultivated in unmodified chemically defined medium during all experiments. The optical density of the reference culture with all ingredients reached 10.9 ± 0.4 at the end of the cultivation.

The average pH of all cultures in this experiment was found to be 9.29 ± 0.04. Notably, almost no variation of the final pH was found in any experiments in this study. pH 9.25 is considered to be the optimum for *S. pasteurii* cultivation and its ATP generation^10^. This alkaline environment is caused by the ureolytic properties of the microorganism itself^32^. Therefore, no negative impacts from alkaline pH on growth was observed.

**Table 1:** Composition of the chemically defined medium.

**Ingredients**	Concentration	Final
	Stock solution (g/L)	Concentration (g/L)
**Main components**		
Glucose monohydrate		11.00
Sodium acetate trihydrate		1.660
K$_{2}$HPO$_{4}$		1.700
Urea		20.00
NaCl		5.000
Ammonia stock solution: 20 mL/L		
$(\hbox {NH}_4)_2\hbox {SO}_4$	374.50	7.490
**Trace elements**		
Iron stock solution: 5 mL/L		
$\hbox {FeCl}_3\cdot 6\hbox {H}_2\hbox {O}$	1	0.005
$\hbox {FeCl}_{2}$	1	0.005
Micronutrient stock solution: 50 mL/L		
$\hbox {MgCl}_2\cdot 6\hbox {H}_2\hbox {O}$	8.54	0.427
$\hbox {MnSO}_4\cdot 4\hbox {H}_2\hbox {O}$	0.56	0.028
$\hbox {ZnSO}_4\cdot 7\hbox {H}_2\hbox {O}$	0.18	0.009
$\hbox {CoSO}_4\cdot 7\hbox {H}_2\hbox {O}$	0.085	0.004
$\hbox {CuSO}_4\cdot 5\hbox {H}_2\hbox {O}$	0.08	0.004
$(\hbox {NH}_4)_6\hbox {Mo}_7\hbox {O}_{24}\cdot 4\hbox {H}_2\hbox {O}$	0.06	0.003
$\hbox {NiCl}_2\cdot 6\hbox {H}_2$O	0.2	0.010
EDTA	0.2	0.010
**Glutamate group**		
L-glutamine (Gln)		1.000 *
Glutamate stock solution: 125 mL/L		
L-histidine (His)	1.2	0.150 *
L-proline (Pro)	5.4	0.675 *
L-arginine (Arg)	1	0.125 *
L-glutamic acid stock solution: 250 mL/L		
L-glutamic acid (Glu)	2	0.500 *
**Aspartate group**		
Aspartate stock solution: 250 mL/L		
L-aspartic acid (Asp)	1.68	0.420
L-isoleucin (Ile)	0.84	0.210
L-threonin (Thr)	0.9	0.225
L-Methionine stock solution: 20 mL/L		
L-methionine (Met)	6.25	0.125
**Serine group**		
Serine stock solution: 20 mL/L		
L-cysteine (Cys)	6.5	0.130
Glycine (Gly)	8.75	0.175
L-serine stock solution: 20 mL/L		
L-serine (Ser)	17	0.340
**Aromatic group**		
L-tyrosine (Tyr)		0.250
Aromatic stock solution: 20 mL/L		
L-phenylalanine (Phe)	13.75	0.275
L-tryptophan (Trp)	2.5	0.050
**Pyruvate group**		
Pyruvate stock solution: 40 mL/L		
L-alanine (Ala)	6	0.240
L-leucine (Leu)	11.875	0.475
L-lysine monohydrate (Lys)	12.356	0.494
L-valine (Val)	8.125	0.325
**Vitamins**		
Vitamin stock solution: 100 mL/L		
Biotin (B7)	0.03	0.003
Thiamin$\cdot$HCl (B1)	0.01	0.001
Nicotinic acid (B3)	1	0.100
Folic acid (B9)	0.01	0.001
Calciumpantothenate (B5)	0.03	0.003

*These components were tripled for the improved chemically defined medium.

Unsurprisingly, no growth was detected without phosphate or trace elements in the medium (Fig. 1a), as phosphate is required for DNA replication and trace elements play manifold functions in bacterial metabolism. No visible growth differences were detected when *S. pasteurii* was cultivated in a medium without ammonia source. Ammonia is described by multiple papers to be mandatory for growth; however, ammonia can be substituted by urea in the medium, as the organism can produce its own ammonia by ureolysis^29,32^. Omitting acetate seemed to slightly impair growth (Fig. 1a). Acetate might be metabolised by *S. pasteurii*; omitting acetate could therefore result in a reduced biomass concentration. This hypothesis will be discussed again at a later point in this study.

Omitting grouped amino acids led to overall growth deceleration (Fig. 1b). The importance of an abundance of amino acids in the culture medium might be caused by the nitrogen assimilation and amino acid synthesis pathways of *S. pasteurii* DSM33. The microorganism is described to neither show glutamine synthetase nor glutamate synthase activity necessary for efficient nitrogen assimilation; it contains a low-affinity glutamate-dehydrogenase though, which results in impaired inorganic nitrogen assimilation^33^. Glutamate and glutamine, the products of inorganic nitrogen assimilation, play an important role for amino acid synthesis, therefore for protein biosynthesis and consequently also for bacterial growth. Addition of amino acids to the culture therefore might allow skipping slow nitrogen assimilation by directly providing amino acids, which resulted in improved growth. Recent classification of genes of *S. pasteurii* NCTC4822 reports a large number of genes involved in amino acid transport and metabolism^12^. These observations and the results shown here may indicate general strong preference for direct amino acid assimilation over *de novo* biosynthesis. Consequently, this would also suggest that a chemically defined medium with only a small number of components may never result in efficient growth as a majority of proteinogenic amino acids have to be provided by the culture medium.

*S. pasteurii* DSM33 did grow without glutamine contrary to other strains used in the literature^30^ (Fig. 1b, L-glutamine is a component of the glutamate group), which may confirm possible differences in nutritional requirements between strains^29^. No growth was detected without the aspartate and serine groups in the medium (Table 1); therefore, amino acids belonging to these groups are necessary for bacterial growth. The auxotrophic deficiencies for amino acids were further specified to be for L-cysteine and L-methionine (Fig. 1c). Both amino acids notably contain sulphur, which was only present as sulphate in the chemically defined medium. L-cysteine is commonly synthesised during sulphur metabolism, while L-methionine is converted from L-cysteine. In order to rule out the possibility, that the auxotrophic deficiencies for both sulphur-containing amino acids were only caused by the inability of *S. pasteurii* to assimilate or reduce sulphate, it was substituted with sulphur, sodium sulphite and sodium sulphide in the culture media in an additional experiment (shake flask cultivation analogue to precultivation steps). However, there was still no growth detectable with sulphur and sodium sulphite (OD < 0.1 after 48 h, see Supplementary Figure S2 online). Cultivation with sodium sulphide was not pursued due to its toxic properties. Therefore, *S. pasteurii* DSM33 can indeed be considered auxotrophic for L-methionine and L-cysteine.

Furthermore, *S. pasteurii* did not grow without supplementing the vitamin stock solution (Fig. 1b). The known auxotrophic deficiences for thiamine and nicotinic acid^29^ can be confirmed in this study (Fig. 1d). However, *S. pasteurii* DSM33 was not auxotrophic for biotin. Knight and Proom described that some *S. pasteurii* strains are indeed auxotrophic for biotin, but not if they are also auxotrophic for nicotinic acid and vice versa. They come to the conclusion, that different *S. pasteurii* strains are either biotin synthesisers or nicotinic acid synthesisers^29^. This observation is supported by the data found here.

Genome analysis of *Sporosarcina pasteurii* strain BNCC337394 (Reference Sequence: NZ_ CP038012.1) indeed indicates a lack of genes responsible for sulphate assimilation and reduction for L-cysteine (*CysB*, *CysC*, *CysP*) and L-methionine biosynthesis (*metB*, *metC*) in gram-positive bacteria as defined by Ferrario et al.^34^. The same is true for genes required for nicotinic acid *de novo* biosynthesis (*NadA*, *NadB*)^35^. Results are not as clear for thiamine, as its biosynthesis consists of numerous steps involving a multitude of genes, which also vary depending on the microorganism^36^. All in all, phenotypic observations can be matched with genetic analysis of a similar strain. The genome of *S. pasteurii* DSM33, which is used in this study, could not be used for comparison as it is not published yet.

Leaving out L-threonine appears to be beneficial for growth (Fig. 1c). However, this was an outlier probably caused by a higher inoculation volume. This result could not be observed again (see Supplementary Figure S3 online). An additional striking observation during this cultivation experiment is a pronounced almost linear growth phase, even at reference conditions (e.g. Fig. 1a, approx. 12 h, occurring from 11 h to 23 h). Similar linear progression is typical for microbial growth under oxygen limiting conditions as the observed growth rate is then a function of the OTR^37^. However, this case can be ruled out, as oxygen measurements using microplates with DO optodes showed no shortage (minimum pO$_2$ detected: 70 %, see Supplementary Figure S4 online). The cause of the growth at limiting rate was determined in the following experiment.

### Improving the chemically defined medium for *S. pasteurii* cultivation

**Figure 2:**
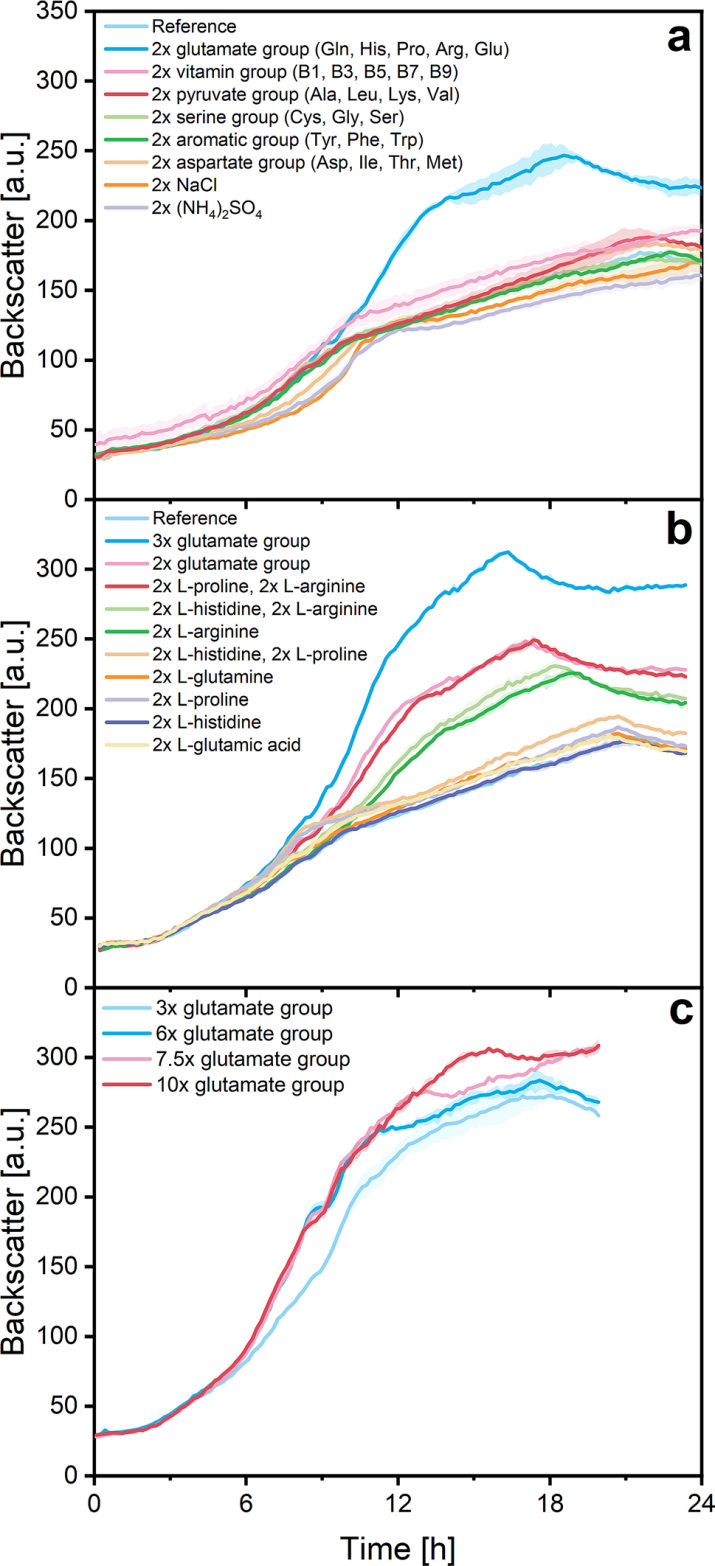
Backscatter data from three different microplate cultivation experiments of *S. pasteurii* in supplemented chemically defined media to determine substrate growth limitations. Illustrated are backscatter data from different growth curves after adding grouped or single components. The reference data corresponds to cultivation in chemically defined medium (Table 1). The cultivation experiments **a**, **b** and **c** were stopped during stationary phase resulting in different total cultivation durations. Average pH of all cultures: 9.29 ± 0.12. Culture conditions: 48-well Flower Well Plate, filling volume 800 $\upmu$L, shaking frequency 1200 rpm, shaking diameter 3 mm and temperature 30 $^\circ$C. Here, arithmetic mean values derived from biological replicates (N = 3) are shown. The error bands depict the standard deviation.

In order to check if the growth at limited rate is caused by consumption of certain medium components, the concentration of grouped components in the medium was doubled in an additional microplate cultivation experiment. Results are illustrated in Fig. 2a, showing clearly better growth with extra amino acids from the glutamate group. However, a limited growth phase is still observable, even though notably shorter (approx. 5 h, occurring from 13 to 18 h). In an additional microplate experiment, the limited growth phase due to substrate limitation was further shortened by tripling all amino acids from the glutamate group in the media (Fig. 2b). Increasing the glutamate amino acid concentration beyond this amount also improved growth, but to a lesser extent (Fig. 2c). The addition of the amino acids L-arginine and L-proline had the greatest impact on shortening the limited growth phase; doubling the concentration of these two amino acids had a similar impact on bacterial growth as doubling the concentration of the entire glutamate group (Fig. 2b), indicating that the lack of these two components is primarily responsible for the pronounced limited growth phase. One question that remains here is why the lack of these amino acids did not limit growth completely but allowed for slower growth. One possible explanation may be that *S. pasteurii* is indeed able to synthesise both amino acids after they are exhausted, but only at a limiting pace. Notably, both amino acids are products from glutamate-sourced reactions. However, extra addition of L-glutamic acid to the medium, which dissociates to glutamate, did not have a positive impact on growth (Fig. 2b). This may indicate that the glutamate-sourced syntheses of L-proline and L-arginine are growth inhibiting and the cause for this limited growth phase. However, this hypothesis has to be proven in further studies. Also, growth improvement could be caused by the presence of additional carbon sources in the form of amino acids from the glutamate group. This possibility is examined in the next section.

With this, growth using a chemically defined medium was strongly improved by 51 % through addition of threefold the glutamate group amino acids. OD600 at the end of cultivation increased from 11.6 ± 0.3 to 17.5 ± 1.2 (Fig. 2b). Additionally, overall growth limiting substrates were detected and the limited growth phase was notably shortened. Therefore, no further medium optimization was performed at this point.

### Detecting useful carbon sources for *S. pasteurii* cultivation

Prior studies claim that *S. pasteurii* is only able to oxidise a limited number of carbohydrates^32^. In order to define which carbon source can be utilized by the organism, an additional microplate cultivation experiment was performed, analogous to the approach of Khani et al.^38^. Glucose as main carbon source in the improved chemically defined medium (Table 1, tripled components marked with asterisk) was substituted with the same amount (10 g/L) of other typical substrates used for fermentation.

**Figure 3:**
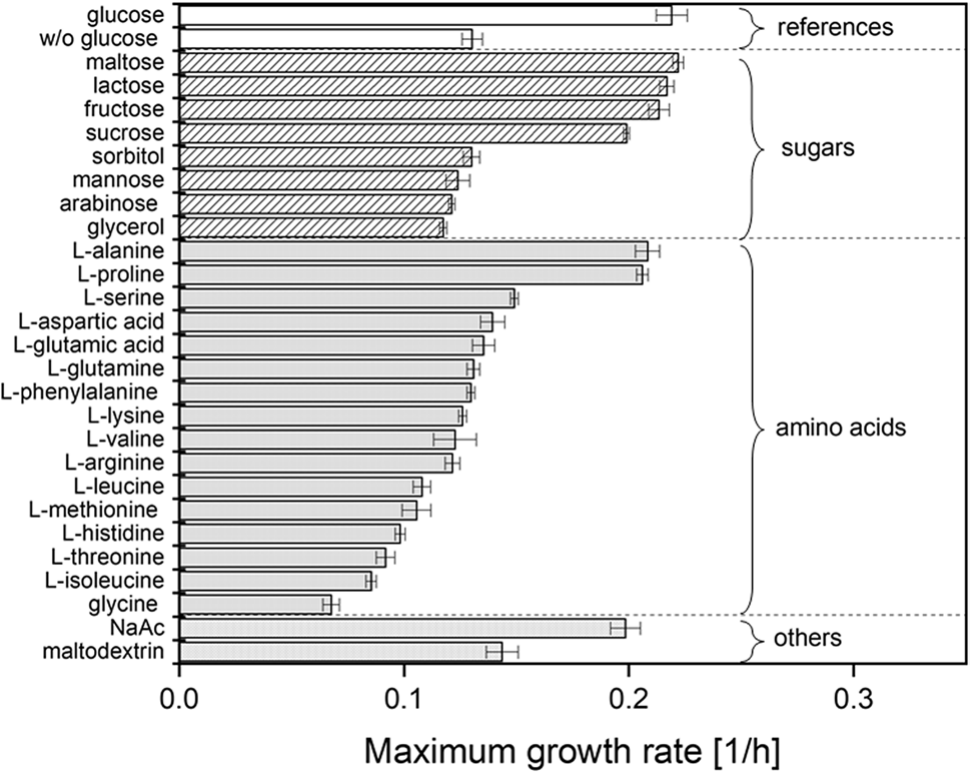
Specific growth rates from microplate cultivation of *S. pasteurii* with substituted main carbon source. All cultivation experiments were performed in improved chemically defined media with modified carbonsource (Table 1, tripled components marked with asterisk). Culture conditions: 48-well Flower Well Plate, filling volume 800 $\upmu$L, shaking frequency 1200 rpm, shaking diameter 3 mm and temperature 30 $^\circ$C. Here, arithmetic mean values derived from biological replicates (N = 3, N = 6 for the references with and without glucose) are shown. The error bars depict the standard deviation.

All backscatter data used for calculation of growth rates can be found in Supplementary Figure S5 online. When using different carbon sources, the lag-phases differed notably despite using the same inoculum. However, no clear systematic reason behind this was found. Consequently, backscatter data suggest that at the end of cultivation some cultures were already in the decline phase while other just entered stationary phase, making comparison based on OD600 at the end of cultivation alone impossible. Therefore, we chose to illustrate the maximum growth rates $\mu$ over the course of the entire cultivation experiments (Fig. 3) for easy comparison. Maximum backscatter data also indicated which carbon sources can be utilized by the organism (see Supplementary Figure S6 online for more information).

*S. pasteurii* did grow moderately even without designated carbon sources at high concentration in this improved medium. Without glucose, the culture reached an optical density of 9.8 ± 0.7. This was probably caused by metabolization of other organic components in the media, e.g. acetate, L-prolin or L-alanine, which proved to be efficient carbon sources similar to glucose regarding growth rate (Fig. 3). The possibility of metabolizing L-proline might indeed contribute to the positive impact from the addition of this amino acid in the form of the glutamate group on bacterial growth in previous experiments (Fig. 2). This has to be elaborated in future studies. Using sodium acetate as main carbon source also allowed for fast growth, again explaining growth impairment after omission in the first experiment (Fig. 1a). Besides these amino acids and acetate, the sugars glucose, maltose, lactose, fructose and sucrose seemed to be beneficial for bacterial growth (Fig. 3). Notably, only mono- and disaccharides, but none of the oligosaccharides and polyols tested (maltodextrin, glycerol, sorbitol), improved growth. Overall, glucose can still be considered as one of the most efficient carbon sources regarding growth rate. These findings also explain the success of already investigated approaches for using waste water as culture medium e.g. using dairy waste containing lactose^39^. These results may also help evaluating other culture media from waste products applicable for cost-efficient industrial scale cultivation of *S. pasteurii*, as e.g. brewery waste waters containing maltose or molasses from sugar refineries containing sucrose.

### Exploiting findings for complex media for *S. pasteurii* cultivation

**Figure 4:**
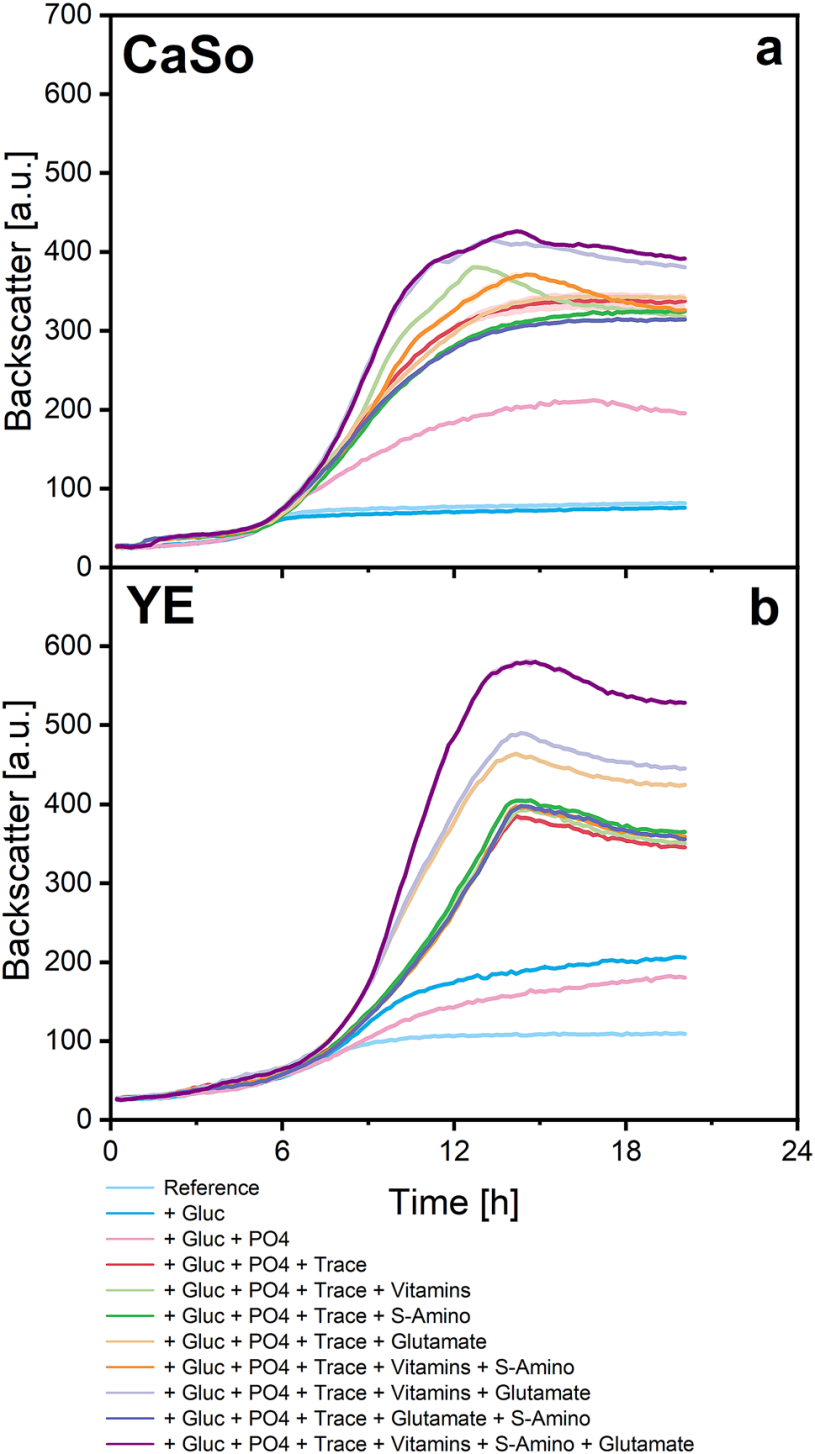
Backscatter data from microplate cultivation of *S. pasteurii* in supplemented complex media. **(a)** Data from casein and soy peptone (CaSo) medium supplementation. **(b)** Data from yeast extract (YE) medium supplementation. Dissolved oxygen (DO) data indicates oxygen limitation between 10 and 14 h for the cultivation with the three supplemented YE media showing the highest improvement (see Supplementary Figure S7 online). This explains slightly linear growth during this period. Components are reported as follow: glucose *Gluc*, K$_2$HPO$_4$ *PO4*, trace elements *Trace*, thiamine-HCl and nicotinic acid *Vitamins*, L-methionine and L-cysteine *S-amino*, triple glutamate group *Glutamate*. Average pH of all cultures: 9.15 ± 0.60. Culture conditions: 48-well Flower Well Plate, filling volume 800 $\upmu$L, shaking frequency 1200 rpm, shaking diameter 3 mm and temperature 30 $^\circ$C. Here, arithmetic mean values derived from biological replicates (N = 2) are shown. The error bands depict the standard deviation.

Findings about nutritional requirements, auxotrophic deficiencies, useful carbon source and further growth limiting substrates in a chemically defined medium of *S. pasteurii* were adapted to supplement frequently used complex media based on peptone and yeast extract (CaSo and YE). Therefore, another microplate cultivation experiment was performed. Components were added according to the concentrations of the improved chemically defined media (Table 1, tripled components marked with asterisk). Results are illustrated in Fig. 4.

In general, both culture media were strongly improved; both media showed the best growth when all described components were supplemented. The optical densities at the end of cultivation were found to be 21.2 ± 0.4 for optimised CaSo medium and 23.2 ± 0.6 for optimised YE medium. Therefore, the optical density in both cases roughly increased fivefold compared to the reference media (see Supplementary Figure S8 online for all OD600 data).

Addition of the trace element solution improved growth on both complex media the most. This comes at no surprise, as peptone and yeast extract media are known to lack trace elements for high cell density cultures^40^. Besides this, the media benefited differently from the additions. For example, adding vitamins to a peptone medium like CaSo did notably improve growth. This effect was not as clear when using the yeast extract medium. Yeast extract is described to already have a high natural vitamin content compared to peptone^41^, explaining lesser impact of further vitamin addition.

We suggest that the supplementations performed here can be adapted to manifold culture media or waste waters used for *S. pasteurii* DSM33 cultivation, allowing for easy process optimization. However, the impact of each component on cultivation performances must be examined for each complex medium and each lot individually, as effects may differ as seen here.

Obviously, supplementing the media results in higher raw material costs; however, the cost effectiveness (Euro/L/OD600) generally improves strongly compared to the unmodified complex media as higher OD600 can be reached (see Supplementary Figure S8 online). Based on calculations regarding the analytical grade chemicals used in this study, the most cost effective medium found here was the CaSo medium with extra glucose, phosphate and trace elements. The price of this medium is about 4.3% higher than the unsupplemented CaSo medium, but results in an increase of the OD600 of about 400%. This example shows that not all supplementations found are necessary or advisable to improve cost effectiveness. In this study, only high quality analytical grade chemicals were used which can be considered as expensive. Food or technical grade chemicals have successfully been tested for *S. pasteurii* cultivation. Their application would additionally reduce costs^21,42^. For large scale industrial applications, media preparation and sterilization must also be considered for cost calculations^20^.

### Analysis of *S. pasteurii* cultivation performance in different media

**Figure 5:**
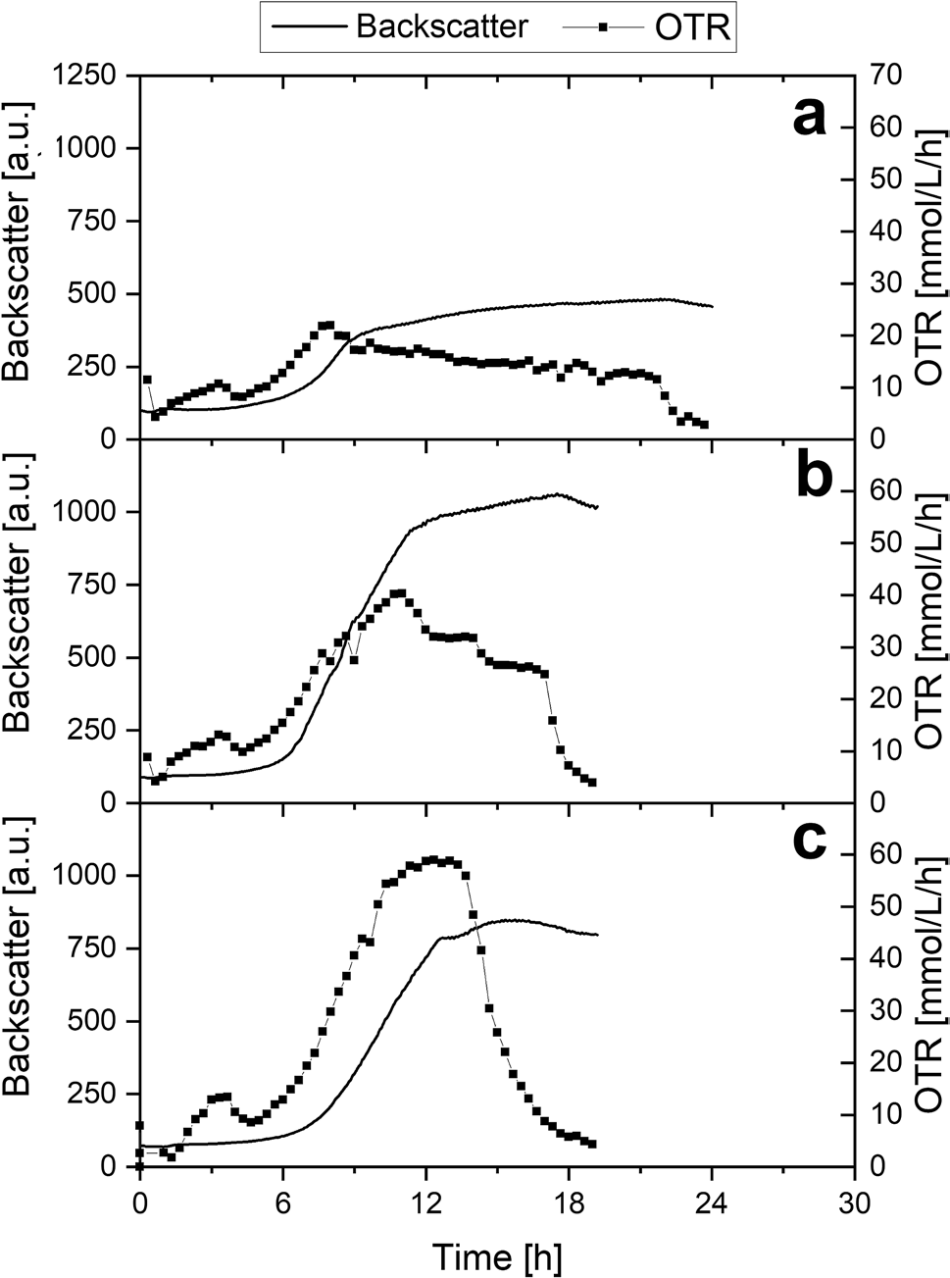
Oxygen transfer rate (OTR) and backscatter of shake flask cultivations of *S. pasteurii* in different media. Backscatter data was generated using the Cell Growth Quantifier system and cannot be compared to backscatter data from microplate cultivation. Cultivation was carried out in **(a)** chemically defined medium, **(b)** improved chemically defined medium **(c)** improved YE medium. Cells were harvested when the OTR decreased to near 0 mmol/L/h. Average pH of all cultures: 9.26 ± 0.09. Culture conditions: 250 mL shake flasks, filling volume 10 mL, shaking frequency 300 rpm, shaking diameter 50 mm and temperature 30 $^\circ$C. Due to the experimental setup, no biological replicates were obtained simultaneously. However, the experiment was repeated with the same results (see Supplementary Figure S10).

Measuring the OTR during cultivation is suitable to identify biological phenomena such as substrate or oxygen limitation, product inhibition or diauxic growth^43^. Non-exponential growth, as observed in this study with unmodified chemically defined medium (e.g. Fig. 1a, approx. 11 h to 23 h), can be a result from these phenomena. Hence, a shake flask cultivation experiment combined with the KuhnerTOM and Cell Growth Quantifier system was carried out in order to analyse cultivation performance and improvement using three media presented in this study (Fig. 5).

During cultivation of *S. pasteurii* in unmodified chemically defined medium (Table 1, Fig. 5a), the OTR as well as the backscatter increase exponentially at the beginning. After 9 h, an almost linear growth phase starts. Simultaneously, the OTR levels off and begins to decrease very slowly until a sudden drop at 22 h. This curve progression is typical for a substrate limitation besides the main carbon source^43^, here probably caused by the lack of glutamate group amino acids in the medium.

For the improved chemically defined medium with a threefold glutamate group amino acid concentration, the maximum OTR of 40 mmol/L/h (Fig. 5b) is notably higher compared to cultivation in unmodified chemically defined medium with 22 mmol/L/h (Fig. 5a). The OTR declines after peaking at approximately 11 h and drops suddenly to almost 0 mmol/L/h after 17 h, indicating complete exhaustion of an essential substrate^43^. This indicates that *S. pasteurii* cultivation is still substrate limited, even though to a much lower degree. Consequently, the chemically defined medium will be optimized further in future studies. Notably, multiple peaks can be detected in the OTR signal, which might indicate metabolization of multiple carbon sources.

When cultivating using the improved YE medium with all supplements as described earlier (Fig. 4b, purple line), the OTR reached an even higher level of 60 mmol/L/h (Fig. 5c) compared to the improved chemically defined medium (Fig. 5b). The plateau between 11 h and 14 h is a characteristic sign of oxygen limitation^43^. After 14 h, the OTR signal drops suddenly, indicating no further limitations when using the improved complex medium.

All OTR curves show a small peak between 2 and 4 h. However, this peak was not as pronounced when the experiment was repeated (see Supplementary Figure S10). One possible explanation for this may be metabolization of multiple carbon sources, resulting in a temporarily higher oxygen demand. This effect will be investigated in further studies.

**Figure 6:**
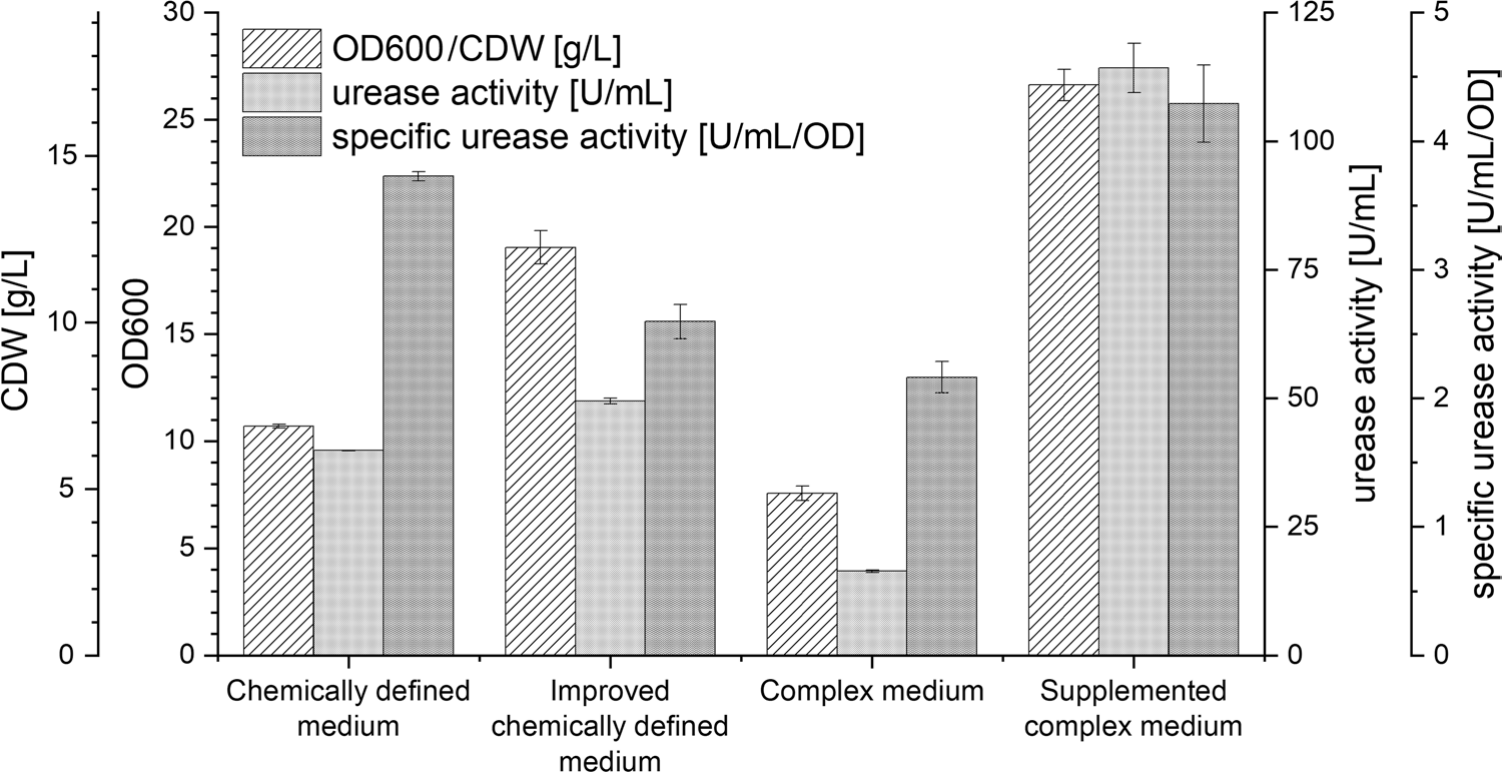
OD600, CDW, urease activity and specific urease activity of shake flask cultivations of *S. pasteurii* in different media, corresponding to the OTR and backscatter data in Fig. 5. CDW was calculated with a predetermined correlation from OD600 data. The chemically defined medium is described in Table 1. The improved chemically defined medium is supplemented with glutamate group amino acids. The complex medium corresponds to the YE medium. OTR and backscatter data from cultivation with the simple YE medium can be found in Supplementary Figure S11. The improved complex medium is supplemented YE medium with glucose, phosphate, trace elements, thiamine-HCl, nicotinic acid, L-methionine, L-cysteine and glutamate group amino acids, according to the concentrations of Table 1, based on the results of Fig. 4b (purple line). Here, arithmetic mean values derived from technical replicates (N = 3) are shown. The error bars depict the standard deviation.

A maximum OD600 of 26.6 ± 0.7 was reached with supplemented YE medium, which is the highest OD600 reached so far in this study. Optical densities at the end of the shake flask cultivation (Fig. 6) are slightly higher to data obtained from microplate cultivations (7 % on average, corresponding to Figs. 1, 2b, 4b). This can be explained by the experimental procedure. Cultures were harvested as soon as the OTR decreased to near 0 mmol/L/h, indicating the exact end of the stationary phase. Decreasing backscatter data from microplate cultivation experiments (Figs. 1, 2b, 4b) shows the end of cultivation already in the decline phase, which is also leading to a reduced optical density.

As stated in the introduction, urease activity is considered to be key for MICP^2^. Urease activity is consequently tackled for optimization by multiple studies^13,15^. The specific urease activity is calculated by dividing the urease activity of 1 mL of the culture by the OD600 at the end of the cultivation. This allows for comparison of the urease activity of the different cultures regardless of the biomass concentration^10,12^. Improving the chemically defined medium results in 60% higher OD600, but the urease activity does not increase accordingly (+ 24 %) (Fig. 6). This leads to a reduced specific urease activity (- 30 %). Supplementing the complex medium resulted in a doubled specific urease activity compared to unsupplemented medium. Therefore, the supplemented complex medium results in the best OD600 without any drawbacks regarding the urease activity.

The urease protein synthesis by *S. pasteurii* is subject to gene regulation^10,12^. However, the exact regulation mechanism is still not fully understood. Exploration of factors influencing the urease activity expression would possibly allow to systematically trigger urease expression which may further improve MICP efficiency. Uncertainty and interfering effects may occur when using complex media for such studies due to their inherent variance^24^. Therefore, using the here developed chemically defined medium may be better suitable for efficient and precise determination of factors influencing the urease synthesis regulation.

## Conclusion

Cultivation of *S. pasteurii* was improved and characterized by using microbioreactor and shake flask online monitoring techniques. This allowed for quickly defining the microorganisms nutritional requirements (phosphate, trace elements), auxotrophic deficiencies (L-methionine, L-cysteine, thiamine, nicotinic acid), usable carbon sources (glucose, maltose, lactose, fructose, sucrose, acetate, L-proline, L-alanine) and further growth limiting substrates (glutamate group amino acids) in a chemically defined medium. In this context, the first chemically defined medium resulting in high OD600 was developed. All components found essential or beneficial for *S. pasteurii* cultivation were supplemented to two simple complex media (containing peptone or yeast extract), increasing the OD at the end of the cultivation by a factor of five. This resulted, to the best of our knowledge, in the highest OD600 of a *S. pasteurii* batch culture published so far.

The here proposed method to supplement media strongly improves media cost effectiveness and make MICP economically more feasible for industrial applications e.g. in the construction industry. Adapting these supplementations is expected to also result in better *S. pasteurii* cultivation performance when using manifold other culture media or waste waters, which is to be elaborated in future studies.

Batch cultivation of *S. pasteurii* in chemically defined and complex media can probably be optimised even further by exactly determine how much of each component is to be supplemented. Microbial urease activity is a prerequisite for efficient MICP and will be optimised accordingly using the media presented in this study. Finally, biocementation experiments will be conducted using bacteria cultivated in the new developed media.

## Methods

### Microorganism and cultivation

*Sporosarcina pasteurii* (DSM33) was obtained from the German Collection of Microorganisms and Cell Culture GmbH (DSMZ, Braunschweig).

Shake flask cultivation for cryo-preservation or precultivation was carried out in 250 mL shake flasks at 30 $^\circ$C at a shaking frequency of 200 rpm, a shaking diameter of 50 mm and a filling volume of 24 mL using a darkened incubator shaker (LT-X, Kuhner AG, Birsfelden). Darkening the cultivation chamber allowed for online monitoring of bacterial growth using a backscatter measurement system (Cell Growth Quantifier, CGQ, aquila biolabs, Baesweiler) with a measuring interval of 20 s.

For cryo-preservation, the strain was cultivated according to the shake flask cultivation steps in CaSo medium with urea, as suggested by DSMZ. Cells were harvested at the end of exponential growth based on visual inspection of backscatter data. After harvesting, 15 % v/v glycerol were added to the culture before freezing it at – 80 $^\circ$C in aliquots of 500 $\upmu$L.

For precultivation, cells from the glycerol stock were thawed and 240 $\upmu$L were added to chemically defined medium, leading to an OD600 (SpectraMax iD3, Molecular Devices, USA) of approximately 0.05 to 0.1. The preculture was then cultivated according to the shake flask cultivation steps. Cells were harvested at the end of the exponential growth phase, centrifuged at 6250 rcf for 5 minutes (Z300K centrifuge, Hermle, Wehingen) and washed with phosphate-buffered saline (10 mM phosphate, Rotifair PBS 7.4, Roth) two times before being used for inoculation for main experiments. Cell washing was performed to prevent carrying over residues from pre-cultivation medium to main cultivation experiments.

Bacterial growth during microplate cultivation was monitored via backscatter quantification at 620 nm using the BioLector I system (m2p-labs, Baesweiler) combined with 48-well baffled microplates with Dissolved Oxygen (DO) optodes (Flower Plates MTP-48-BO 1, m2p-labs, Baesweiler) at 30 $^\circ$C with a shaking diameter of 3 mm, a shaking frequency of 1200 rpm and a filling volume of 800 $\upmu$L. This results in a maximum OTR of 75 mmol/L/h according to the microplate manufacturer (Flower Plate Technical Data Sheet, m2p-labs, Baesweiler, OTR determined with aqueous sulfite oxidation system, results may vary based on temperature and osmolarity of the solution). The detector sensitivity of the system was set to a gain 20 in the software. Humidity control of the BioLector I system was activated (relative humidity $\ge$ 85 %) in order to reduce evaporation. Sterile culture media were inoculated with 1 % v/v washed inoculum from precultivation, and thoroughly mixed before being added in the microplate wells. The microplates were sealed with a sterile gas permeable sealing foil (Sealing foil basic F-GP-10, m2p-labs, Baesweiler) allowing for sufficient oxygen transfer and reduced evaporation^44^. When possible, the cultivation was stopped at stationary phase, which is indicated by the backscatter data or by a DO signal close to 100 %. The maximum growth rate was determined based on backscatter data using the BioLection software. For this, the maximum of the specific growth rates over the entire cultivation period was picked. The specific growth rates were calculated by the following formula, using an average of 50 data points to determine the differential quotient (according to Instruction Manual, BioLector I, m2p-labs, Baesweiler):

1 $$\begin{aligned} \mu = \frac{1}{Backscatter(t)} \times \frac{d(Backscatter(t))}{dt} \end{aligned}$$

Cellular respiration was analysed via online-monitoring of the OTR during shake-flask cultivation using the KuhnerTOM system (Kuhner, Birsfelden). Shake flask cultivation was carried out in 250 mL shake flasks with a filling volume of 10 mL at 30 $^\circ$C at a shaking frequency of 300 rpm and a shaking diameter of 50 mm. The KuhnerTOM system positioned at the top of the cultivation chamber was combined with the backscatter measurement system (CGQ, aquila biolabs, Baesweiler) below individual shake flasks to match OTR data to bacterial growth.

At the end of the cultivation experiments, optical density at a wavelength of 600 nm was measured using a microplate reader (SpectraMax iD3, Molecular Devices, USA. 96 well black microplate $\upmu$CLEAR, Greiner Bio-One, Kremsmünster). Samples were diluted when necessary according to the Beer–Lambert law. Cell dry weight (CDW) was calculated by the formula $CDW [g/L] = 0.6434 \, g/L \times OD600$ (R$^2$ = 0.9749). The correlation between OD600 and CDW was obtained by a procedure similarly described by Kensy et al.^31^ (see Supplementary File S13). The pH value of the fermentation broth was measured using a portable pH meter (Seven2go pH Meter, Mettler Toledo, Giessen). For shake flask experiments, urease activity was determined by a conductivity assay established by Whiffin^10^. A standard curve was generated using urease (Sigma aldrich, 666133-10KU) with known enzymatic activity (see Supplementary Figure S14). The manufacturer defines one unit as the amount of enzyme that will release 1 $\upmu$mol of ammonia from urea per min at 25 $^{\circ }$C, pH 7.0.

### Media and solutions

CaSo medium, also called Medium 220 by DSMZ, contains 15 g/L peptone from casein (Roth, 258268767), 5 g/L peptone from soy (Roth, 098266388) and 5 g/L NaCl, and is mixed after autoclaving with 1:6 parts of sterile filtrated 120 g/L urea solution, resulting in a final concentration of 20 g/L urea in the medium. Yeast extract medium (YE), used as an example for improving complex media, consists of 20 g/L yeast extract (Roth, 175228598) and 20 g/l urea. When additional ingredients were added to the complex media, the media were titrated to a pH of 7 with NaOH or HCl if necessary and sterile filtered afterwards. In this case, the media were not autoclaved.

The chemically defined medium used in this study is based on an adaption of Poolman medium^45^ by Müller et al.^27^ and adapted again in multiple iteration steps for our purposes (see Supplementary Table S12 online). All ingredients are listed in Table 1. Components are grouped based on Müller et al.^27^. The chemically defined medium consists of multiple stock solutions; the L-glutamic acid, L-methionine, L-serine and vitamin stock solution were freshly made for each cultivation. After preparation, the medium was titrated to a pH of 7 with NaOH or HCl and sterile filtered afterwards.

When media were modified for main experiments, the components were either omitted or added according to the concentrations described in Table 1. All chemicals used in this study were of analytical grade (Roth, Sigma-Aldrich, AppliChem). All substances were diluted in demineralized water.

## Supplementary Information

Supplementary Information.

## Data Availability

The datasets generated during and/or analysed during the current study are available from the corresponding author on reasonable request.
