# Publisher Correction: Revealing nutritional requirements of MICP-relevant *Sporosarcinapasteurii* DSM33 for growth improvement in chemically defined and complex media

**DOI:** 10.1038/s41598-022-06174-y

**Published:** 2022-02-01

**Authors:** Frédéric M. Lapierre, Jakob Schmid, Benjamin Ederer, Nina Ihling, Jochen Büchs, Robert Huber

**Affiliations:** 1 Munich University of Applied Sciences, 80335 Munich, Germany; 2 Chair of Biochemical Engineering (AVT.BioVT), RWTH Aachen University, 52074 Aachen, Germany

Correction to: *Scientific Reports* 10.1038/s41598-020-79904-9, Published online 31 December 2020

The original version of this Article contained errors in the References 12, 13, 23, 24, 25, 26, 27, 29, 30, 34, 36, 37, 42, 43, and 44, which were incorrectly given as:

12. Ma, L., Pang, A.-P., Luo, Y., Lu, X. & Lin, F. Beneficial factors for biomineralization by ureolytic bacterium *Sporosarcina pasteurii*. *Microb. Cell Fact.* **19**, 12. https://doi.org/10.3390/su121562810 (2020).

13. Omoregie, A. I., Khoshdelnezamiha, G., Senian, N., Ong, D. E. L. & Nissom, P. M. Experimental optimisation of various cultural conditions on urease activity for isolated *Sporosarcina pasteurii* strains and evaluation of their biocement potentials. *Ecol. Eng.* **109**, 65–75. https://doi.org/10.3390/su121562811 (2017).

23. Aller, K. et al*.* Nutritional requirements and media development for lactococcus lactis il1403. *Appl. Microbiol. Biotechnol.* **98**, 5871–5881. https://doi.org/10.3390/su121562819 (2014).

24. Diederichs, S. et al*.* Phenotyping the quality of complex medium components by simple online-monitored shake flask experiments. *Microb. Cell Fact.* **13**, 149. https://doi.org/10.1016/j.ecoleng.2009.02.0060 (2014).

25. Kim, Y. J. et al*.* Development of a chemically defined minimal medium for the exponential growth of leuconostoc mesenteroides atcc8293. *J. Microbiol. Biotechnol.* **22**, 1518–1522. https://doi.org/10.1016/j.ecoleng.2009.02.0061 (2012).

26. Chervaux, C., Ehrlich, S. . D. & Maguin, E. Physiological study of lactobacillus delbrueckii subsp. bulgaricus strains in a novel chemically defined medium. *Appl. Environ. Microbiol.* **66**, 5306–5311. https://doi.org/10.1016/j.ecoleng.2009.02.0062 (2000).

27. Müller, J., Beckers, M., Mußmann, N., Bongaerts, J. & Büchs, J. Elucidation of auxotrophic deficiencies of bacillus pumilus dsm 18097 to develop a defined minimal medium. *Microb. Cell Fact.* **17**, 106. https://doi.org/10.1016/j.ecoleng.2009.02.0063 (2018).

29. Knight, B. C. J. G. & Proom, H. A comparative survey of the nutrition and physiology of mesophilic species in the genus bacillus. J. Gen. Microbiol. 4, 508–538. https://doi.org/10.1016/j.ecoleng.2009.02.0064 (1950).

30. Klein, M., Kaltwasser, H. & Jahns, T. Isolation of a novel, phosphate-activated glutaminase from *Bacillus pasteurii*. *FEMS Microbiol. Lett.* **206**, 63–67. https://doi.org/10.1016/j.ecoleng.2009.02.0065 (2002).

34. Ferrario, C. et al*.* Exploring amino acid auxotrophy in bifidobacterium bifidum prl2010. *Frontiers in microbiology* **6**, 1331. https://doi.org/10.1016/j.ecoleng.2009.02.0068 (2015).

36. Jurgenson, C. T., Begley, T. P. & Ealick, S. E. The structural and biochemical foundations of thiamin biosynthesis. *Annu. Rev. Biochem.* **78**, 569–603. https://doi.org/10.1007/s42729-019-00121-z0 (2009).

37. Maier, U. & Büchs, J. Characterisation of the gas–liquid mass transfer in shaking bioreactors. *Biochem. Eng. J.* **7**, 99–106. https://doi.org/10.1007/s42729-019-00121-z1 (2001).

42. Omoregie, A. I., Palombo, E. A., Ong, D. E. & Nissom, P. M. Biocementation of sand by *Sporosarcina pasteurii* strain and technical-grade cementation reagents through surface percolation treatment method. *Constr. Build. Mater.* **228**, 116828. https://doi.org/10.1007/s42729-019-00121-z6 (2019).

43. Anderlei, T. & Büchs, J. Device for sterile online measurement of the oxygen transfer rate in shaking flasks. *Biochem. Eng. J.* **7**, 157–162. https://doi.org/10.1007/s42729-019-00121-z7 (2001).

44. Sieben, M., Giese, H., Grosch, J.-H., Kauffmann, K. & Büchs, J. Permeability of currently available microtiter plate sealing tapes fail to fulfil the requirements for aerobic microbial cultivation. *Biotechnol. J.* **11**, 1525–1538. https://doi.org/10.1007/s42729-019-00121-z8 (2016).

The correct references are listed below:

12. Ma, L., Pang, A.-P., Luo, Y., Lu, X. & Lin, F. Beneficial factors for biomineralization by ureolytic bacterium *Sporosarcina pasteurii*. *Microb. Cell Fact.* **19**, 12. https://doi.org/10.1186/s12934-020-1281-z (2020).

13. Omoregie, A. I., Khoshdelnezamiha, G., Senian, N., Ong, D. E. L. & Nissom, P. M. Experimental optimisation of various cultural conditions on urease activity for isolated *Sporosarcina pasteurii* strains and evaluation of their biocement potentials. *Ecol. Eng.* **109**, 65–75. https://doi.org/10.1016/j.ecoleng.2017.09.012 (2017).

23. Aller, K. et al*.* Nutritional requirements and media development for lactococcus lactis il1403. *Appl. Microbiol. Biotechnol.* **98**, 5871–5881. https://doi.org/10.1007/s00253-014-5641-7 (2014).

24. Diederichs, S. et al*.* Phenotyping the quality of complex medium components by simple online-monitored shake flask experiments. *Microb. Cell Fact.* **13**, 149. https://doi.org/10.1186/s12934-014-0149-5 (2014).

25. Kim, Y. J. et al*.* Development of a chemically defined minimal medium for the exponential growth of leuconostoc mesenteroides atcc8293. *J. Microbiol. Biotechnol.* **22**, 1518–1522. https://doi.org/10.4014/jmb.1205.05053 (2012).

26. Chervaux, C., Ehrlich, S. . D. & Maguin, E. Physiological study of lactobacillus delbrueckii subsp. bulgaricus strains in a novel chemically defined medium. *Appl. Environ. Microbiol.* **66**, 5306–5311. https://doi.org/10.1128/AEM.66.12.5306-5311.2000 (2000).

27. Müller, J., Beckers, M., Mußmann, N., Bongaerts, J. & Büchs, J. Elucidation of auxotrophic deficiencies of bacillus pumilus dsm 18097 to develop a defined minimal medium. *Microb. Cell Fact.* **17**, 106. https://doi.org/10.1186/s12934-018-0956-1 (2018).

29. Knight, B. C. J. G. & Proom, H. A comparative survey of the nutrition and physiology of mesophilic species in the genus bacillus. *J. Gen. Microbiol.* **4**, 508–538. https://doi.org/10.1099/00221287-4-3-508 (1950).

30. Klein, M., Kaltwasser, H. & Jahns, T. Isolation of a novel, phosphate-activated glutaminase from *Bacillus pasteurii*. *FEMS Microbiol. Lett.* **206**, 63–67. https://doi.org/10.1111/j.1574-6968.2002.tb10987.x (2002).

34. Ferrario, C. et al*.* Exploring amino acid auxotrophy in bifidobacterium bifidum prl2010. *Frontiers in microbiology* **6**, 1331. https://doi.org/10.3389/fmicb.2015.01331 (2015).

36. Jurgenson, C. T., Begley, T. P. & Ealick, S. E. The structural and biochemical foundations of thiamin biosynthesis. *Annu. Rev. Biochem.* **78**, 569–603. https://doi.org/10.1146/annurev.biochem.78.072407.102340 (2009).

37. Maier, U. & Büchs, J. Characterisation of the gas–liquid mass transfer in shaking bioreactors. *Biochem. Eng. J.* **7**, 99–106. https://doi.org/10.1016/S1369-703X(00)00107-8 (2001).

42. Omoregie, A. I., Palombo, E. A., Ong, D. E. & Nissom, P. M. Biocementation of sand by *Sporosarcina pasteurii* strain and technical-grade cementation reagents through surface percolation treatment method. *Constr. Build. Mater.* **228**, 116828. https://doi.org/10.1016/j.conbuildmat.2019.116828 (2019).

43. Anderlei, T. & Büchs, J. Device for sterile online measurement of the oxygen transfer rate in shaking flasks. *Biochem. Eng. J.* **7**, 157–162. https://doi.org/10.1016/s1369-703x(00)00116-9 (2001).

44. Sieben, M., Giese, H., Grosch, J.-H., Kauffmann, K. & Büchs, J. Permeability of currently available microtiter plate sealing tapes fail to fulfil the requirements for aerobic microbial cultivation. *Biotechnol. J.* **11**, 1525–1538. https://doi.org/10.1002/biot.201600054 (2016).

The original Article has been corrected.

