# Supplementary material for: Revealing nutritional requirements of MICP-relevant Sporosarcina pasteurii DSM33 for growth improvement in chemically defined and complex media
Source: Sci Rep. 2020 Dec 31;10:22448. doi: 10.1038/s41598-020-79904-9 (PMC7775470; doi:10.1038/s41598-020-79904-9)
Supplement: Supplementary file 1 — Supplementary Information. [file 41598_2020_79904_MOESM1_ESM.pdf]

Supplementary information: Revealing nutritional requirements of MICP-relevant *Sporosarcina pasteurii* DSM33 for growth improvement in chemically defined and complex media

Frédéric M. Lapierre[1,*], Jakob Schmid[1], Benjamin Ederer[1], Nina Ihling[2], Jochen Büchs[2] and Robert Huber[1]
[1]Munich University of Applied Sciences, Munich, 80335 Germany
[2]Chair of Biochemical Engineering (AVT.BioVT), RWTH Aachen University, Aachen, 52074 Germany
*frederic.lapierre@hm.edu

Supplementary file 1 – Experiment summary

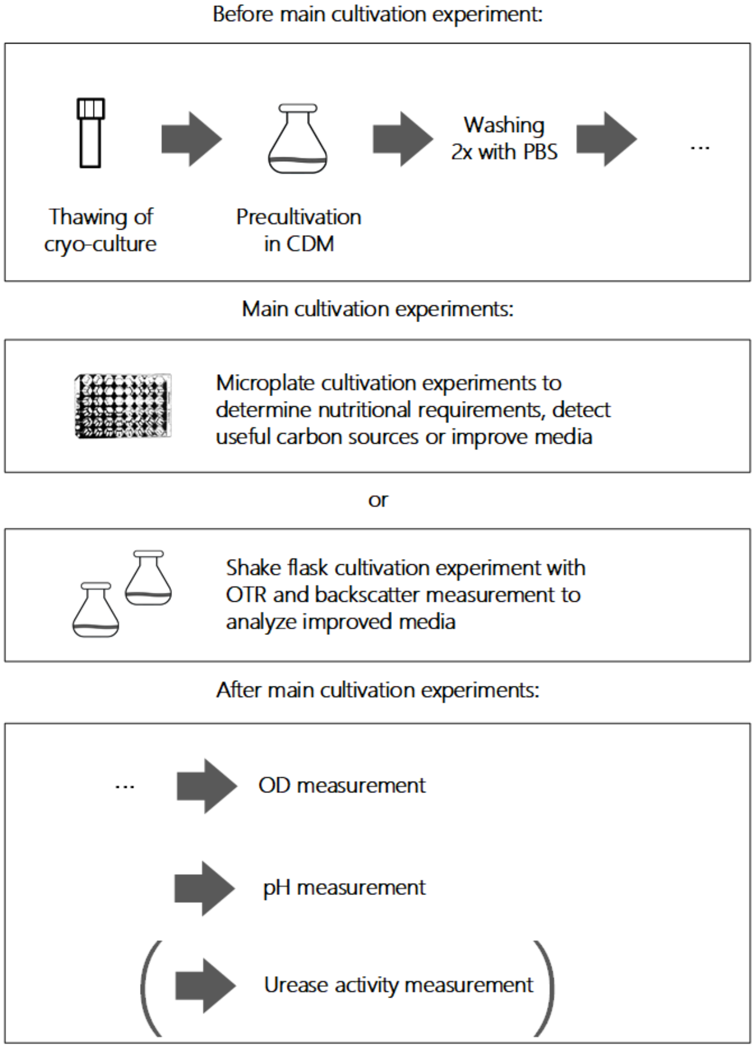

Figure S1. Overview of cultivation experiments to develop high biomass concentration media. Details can be found in the "Material and methods" section. Chemically defined medium *CDM*, phosphate-buffered saline *PBS*.

Supplementary file 2 – Influence of sulphur sources on *S. pasteurii* cultivation

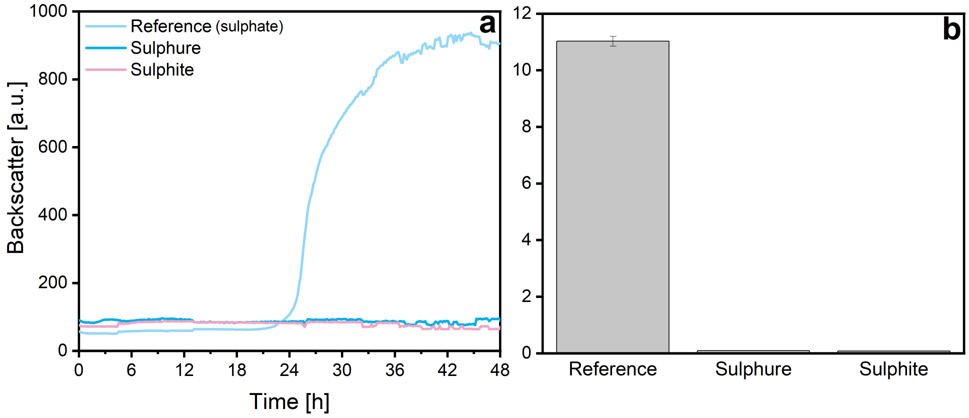

Figure S2. **(a)** Backscatter and **(b)** OD600 data of shake flask cultivations of *S. pasteurii* in chemically defined media with substituted sulphur sources. The reference data corresponds to cultivation in chemically defined medium with sulphate (Table 1). The amino acids L-methionine and L-cysteine were omitted from the other cultivation media and sulphate was substituted with sulphur and sulphite with the same molar concentrations as sulphate in the reference medium. No growth was detectable. Culture conditions: 250 mL shake flasks, filling volume 20 mL, shaking frequency 200 rpm, shaking diameter 50 mm and temperature 30 °C. For **(b)**, arithmetic mean values derived from technical replicates (N = 3) are shown. The error bars depict the standard deviation.

Supplementary file 3 – Influence of L-threonine omission on *S. pasteurii* cultivation

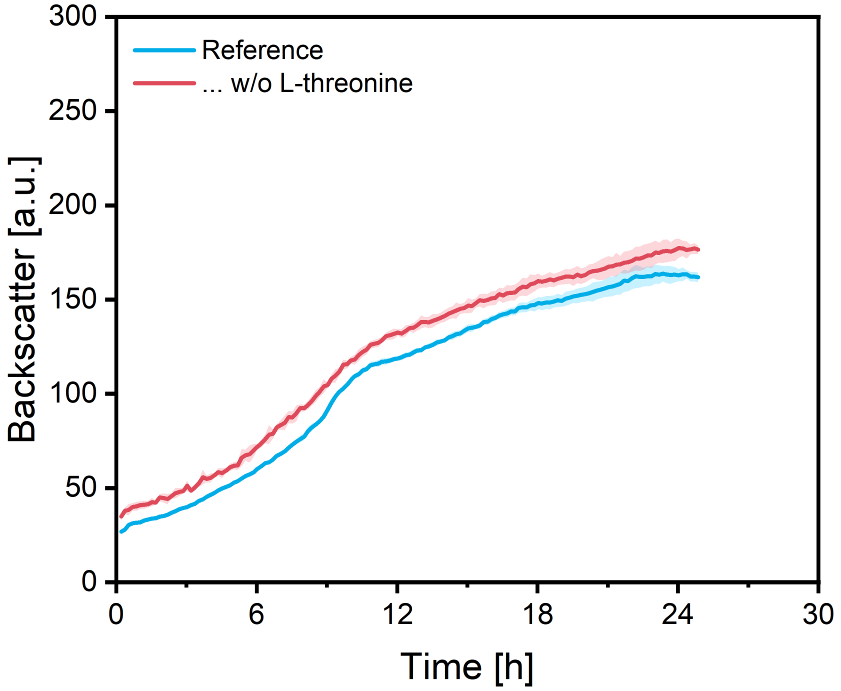

Figure S3. Backscatter data from microplate cultivation of *S. pasteurii* to replicate the effect of L-threonine in chemically defined medium. As in Figure 1, backscatter of cultivation without L-threonine is slightly higher than cultivation at reference conditions. However, the curve progression appears to be identical. The reference data corresponds to cultivation in chemically defined medium (Table 1). Average pH of all cultures: 9.12 ± 0.01. Culture conditions: 48-well Flower Well Plate, filling volume 800 μL, shaking frequency 1200 rpm, shaking diameter 3 mm and temperature 30 °C. Here, arithmetic mean values derived from biological replicates (N = 6) are shown. The error bands depict the standard deviation.

Supplementary file 4 – Dissolved oxygen data of *S. pasteurii* cultivation with chemically defined medium

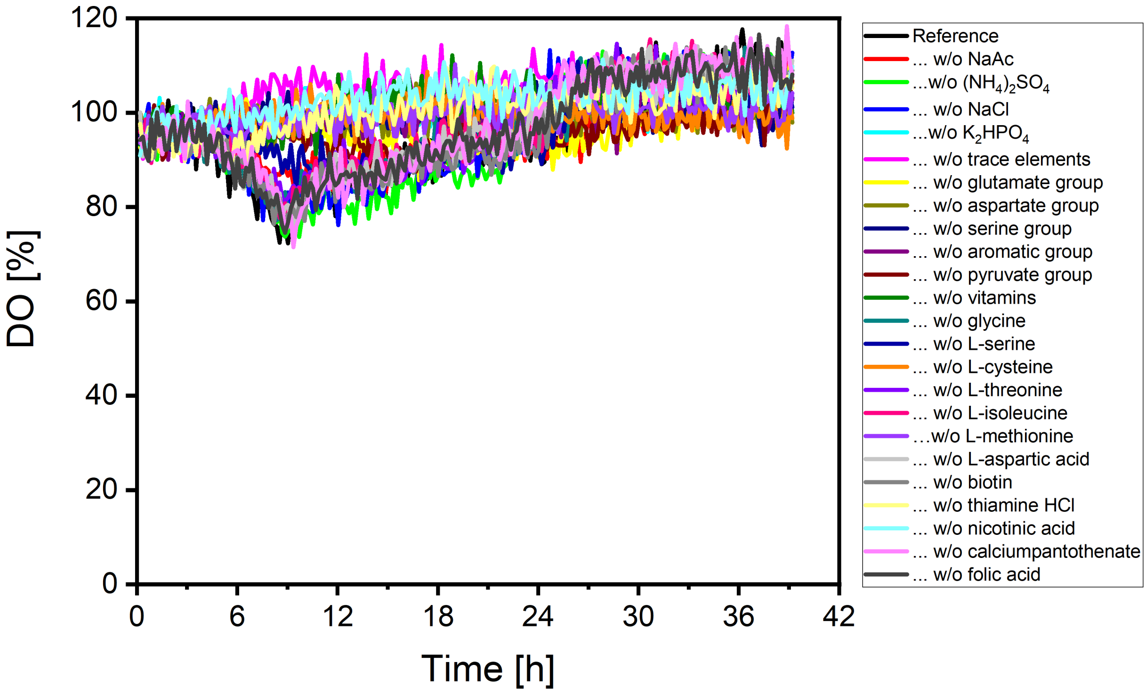

Figure S4. DO data from a microplate cultivation of *S. pasteurii* in modified chemically defined media, corresponding to backscatter data illustrated in Figure 1. As stated in the manuscript, DO data showed no oxygen shortage. Culture conditions: 48-well Flower Well Plate, filling volume 800 μL, shaking frequency 1200 rpm, shaking diameter 3 mm and temperature 30 °C. Here, arithmetic mean values derived from biological replicates (N = 2, N = 1 for w/o vitamins) are shown.

Supplementary file 5 – Backscatter cultivation data for different main carbon sources

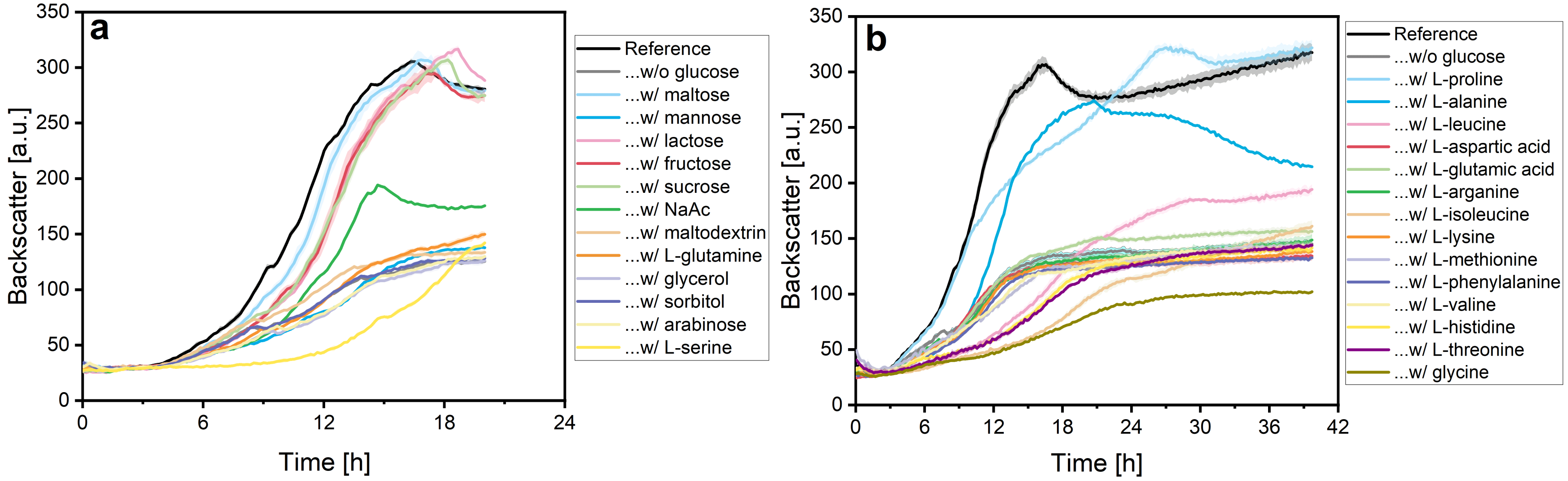

Figure S5. Backscatter data from two microplate cultivation experiments of *S. pasteurii* in improved chemically defined media with substituted main carbon source. Illustrated are backscatter data from different growth curves after substituting glucose as main carbon sources. The reference data corresponds to cultivation in improved chemically defined medium (Table 1, tripled components marked with asterisk). Average pH of all cultures: 9.24 ± 0.05. Culture conditions: 48-well Flower Well Plate, filling volume 800 μL, shaking frequency 1200 rpm, shaking diameter 3 mm and temperature 30 °C. Here, arithmetic mean values derived from biological replicates (N = 3) are shown. The error bands depict the standard deviation.

Supplementary file 6 – Relation between maximum growth rate, maximum backscatter and OD600 at the end of the cultivation for different carbon sources

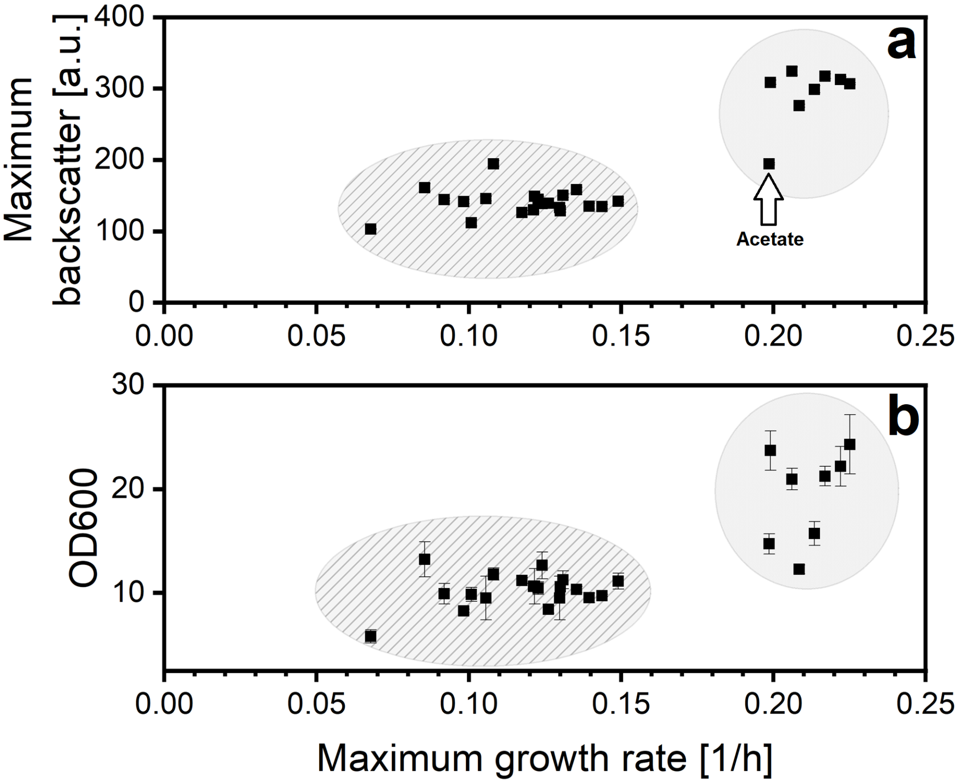

Figure S6. Relation of maximum growth rate to **(a)** maximum backscatter and **(b)** OD600 at the end of the cultivation, corresponding to maximum growth rate data illustrated in Figure 3. Culture conditions: 48-well Flower Well Plate, filling volume 800 μL, shaking frequency 1200 rpm, shaking diameter 3 mm and temperature 30 °C. Here, arithmetic mean values derived from biological replicates (N = 3, N = 6 for the references with and without glucose) are shown. The error bars depict the standard deviation.

As stated in the manuscript, OD600 from cultures showing improved growth scatters between 12.3 and 24.3 (Figure S6b), as some cultures were already in the decline phase while other just entered stationary phase at the end of cultivation (Figure S5). Therefore, using the OD600 at the end of the cultivation as an indicator if a certain carbon source can be metabolized by *S. pasteurii* would have been misleading. However, focussing on the maximum backscatter of each culture allows for comparison without taking the different growth phases into account. Cultures with high maximum backscatter also have a high maximum growth rate (Figure S6a), showing two clearly distinct groups and allowing for summarizing the observed backscatter curves illustrated in Figure S5.

One exception though is the cultivation of *S. pasteurii* with acetate as carbon source. Using acetate as carbon source led to fast growth, but not to a high maximum backscatter (Figure S6a). This is probably caused by different metabolic pathways. Acetyl-CoA is the starting point for the citric acid cycle. Acetate can be directly converted to acetyl-CoA while consuming ATP. Contrary to this, the breakdown of sugars as glucose by glycolysis results in pyruvat, which is then converted to acetyl-CoA, releasing ATP. This process can be seen as overall more energetically profitable for the microorganism, allowing for more efficient growth and therefore higher biomass concentration.

Supplementary file 7 – Dissolved oxygen data of *S. pasteurii* cultivation in supplemented complex media

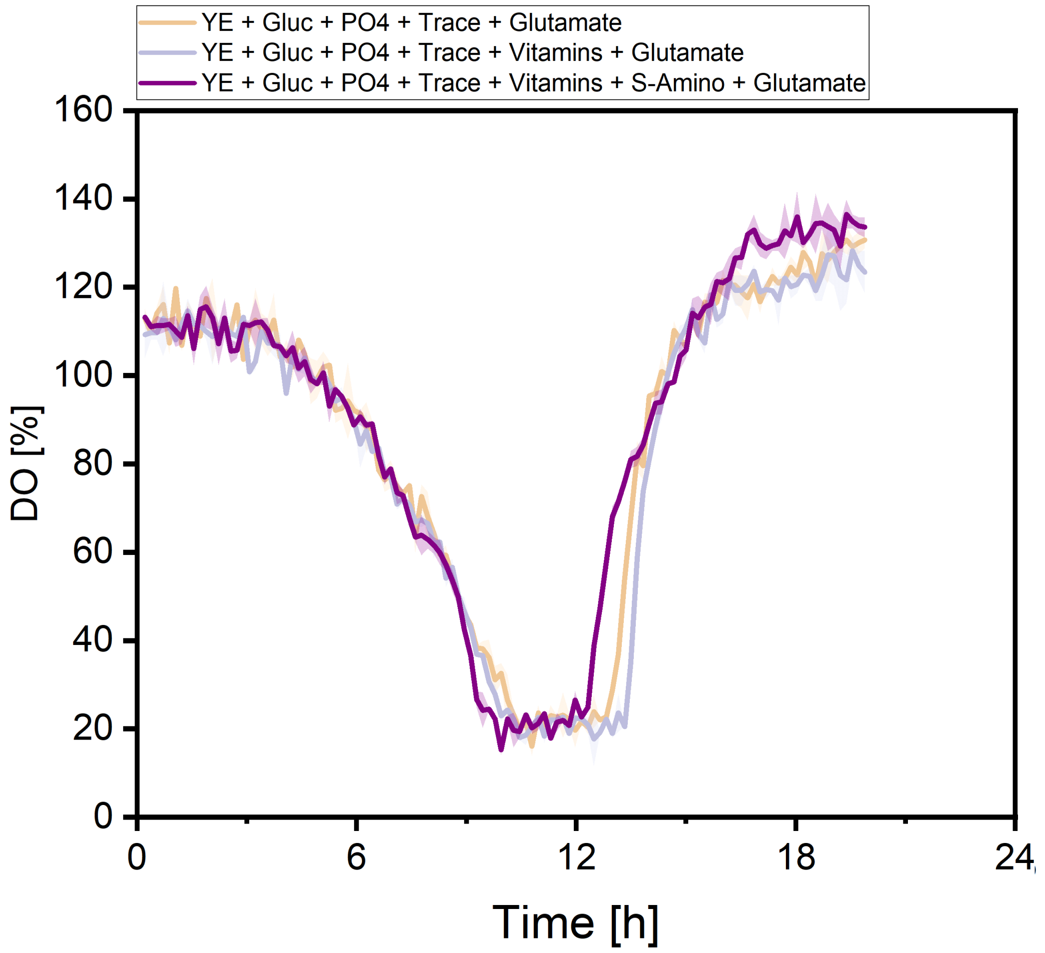

Figure S7. DO data from a microplate cultivation of *S. pasteurii* in supplemented complex media, corresponding to backscatter data illustrated in Figure 4. As stated in the manuscript, oxygen limitation was observed between 10 and 14 h, which can be determined from a very typical plateaued DO curve. Oxygen limitation is normally also indicated by DO measurements equal to zero. However, measurements with DO optodes can be affected by fermentation media, media salinity or product molecules (Instruction Manual, BioLector I, m2p-labs, Baesweiler), which results in a data shift as seen here. Culture conditions: 48-well Flower Well Plate, filling volume 800 μL, shaking frequency 1200 rpm, shaking diameter 3 mm and temperature 30 °C. Here, arithmetic mean values derived from biological replicates (N = 2) are shown. The error bands depict the standard deviation.

Supplementary file 8 – Cost effectiveness of supplemented media

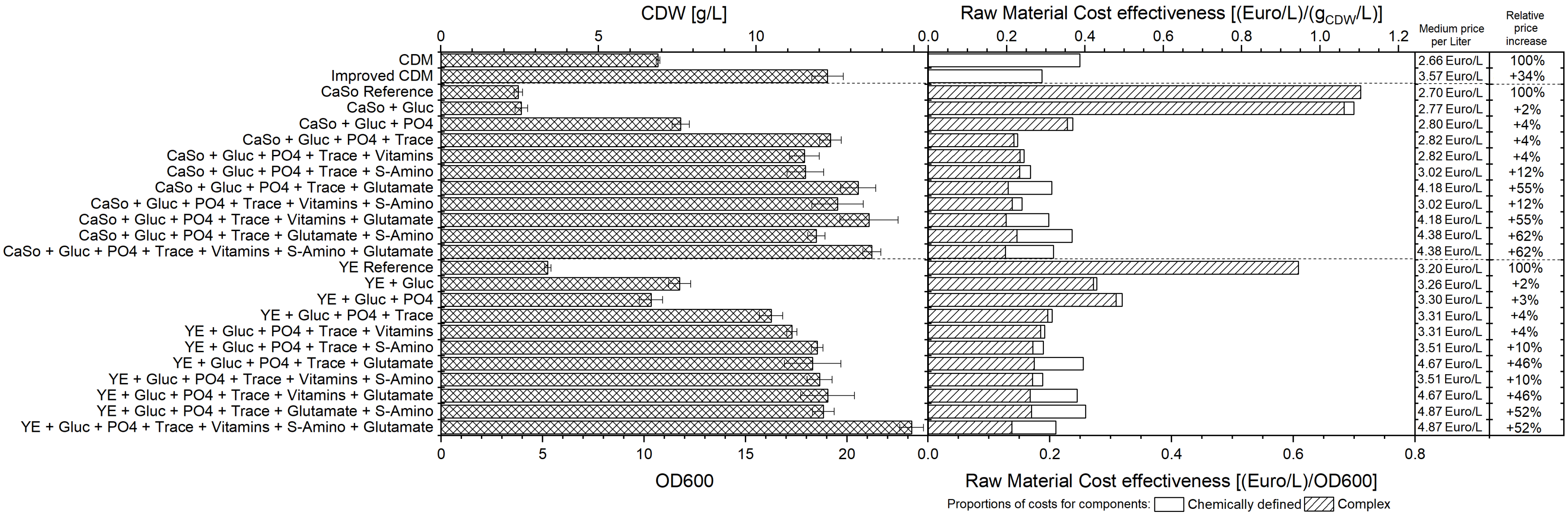

Figure S8. OD600/CDW at the end of cultivation, cost effectiveness, total net media costs and relative media costs for different media. The data corresponds to the microplate cultivation experiment in Figure 4, with the exception of the data for the chemically defined media (*CDM*), which correspond to Figure 5. Ingredients costs per kg are listed in Supplementary File S9. Components are reported as follow: glucose *Gluc*, K_2HPO_4 *PO4*, trace elements *Trace*, thiamine-HCl and nicotinic acid *Vitamins*, L-methionine and L-cysteine *S-amino*, triple glutamate group *Glutamate*. Here, arithmetic mean values are shown. The error bars depict the standard deviation. Cultivations were performed in duplicates (mean values calculated from biological replicates), with the exception of CDM cultivations, which were single experiments with three OD600 measurements (mean values calculated from technical replicates). Culture conditions for complex media: 48-well Flower Well Plate, filling volume 800 μL, shaking frequency 1200 rpm, shaking diameter 3 mm and temperature 30 °C. Culture conditions for CDM: 300 mL shake flasks, filling volume 10 mL, shaking frequency 300 rpm, shaking diameter 50 mm and temperature 30 °C.

As stated in the manuscript, analytical grade chemical net prices were applied for the cost calculations. Additionally, bulk orders for large scale applications would reduce costs. No quantity discount was included in the calculations made here. However, we assume that relative price differences of the media are true nonetheless.

Supplementary file 9 – Net prices of media components

Table S9. Net prices for chemicals used for calculations of media costs, based on data from www.carlroth.com from end of 2019.

Ingredient	Euro/kg
Glucose	6.36
Sodium acetate	11.96
$(NH_4)_2SO_4$	8.37
K_2HPO_4	22.36
Urea	37.80
NaCl	4.00
$MgCl{\cdot}6H_2O$	26.96
$MnCl_2$	56.90
$FeCl_3{\cdot}6H_2O$	36.90
$FeCl_2$	49.56
$ZnSO_4{\cdot}7H_2O$	23.96
$CoSO_4{\cdot}7H_2O$	51.60
$CuSO_4{\cdot}5H_2O$	8.90
$(NH_4)_6Mo_7O_24{\cdot}4H_2O$	95.60
$NiCl_2{\cdot}6H_2O$	51.60
EDTA	57.12
L-alanine	374.00
L-arginine	125.90
L-aspartic acid	110.90
L-cysteine	1,292.50
L-glutamic acid	82.50
Glycine	191.60
L-histidine	327.50
L-isoleucine	325.00
L-leucine	275.00
L-lysine	598.00
L-methionine	258.00
L-phenylalanine	255.00
L-proline	227.00
L-serine	259.00
L-valine	199.00
L-threonine	205.80
L-tryptophan	489.00
L-gyrosine	285.00
L-glutamine	193.00
Biotin	14,970.00
Nicotinic acid	238.00
Calciumpantothenate	133.40
Thiamine·HCl	327.00
Folic acid	1,032.00
Peptone from caseine	99.80
Peptone from soy	86.00
Yeast extract	122.00

Supplementary file 10 – Repeated experiment: OTR and backscatter of shake flask cultivation of *S. pasteurii* in different media

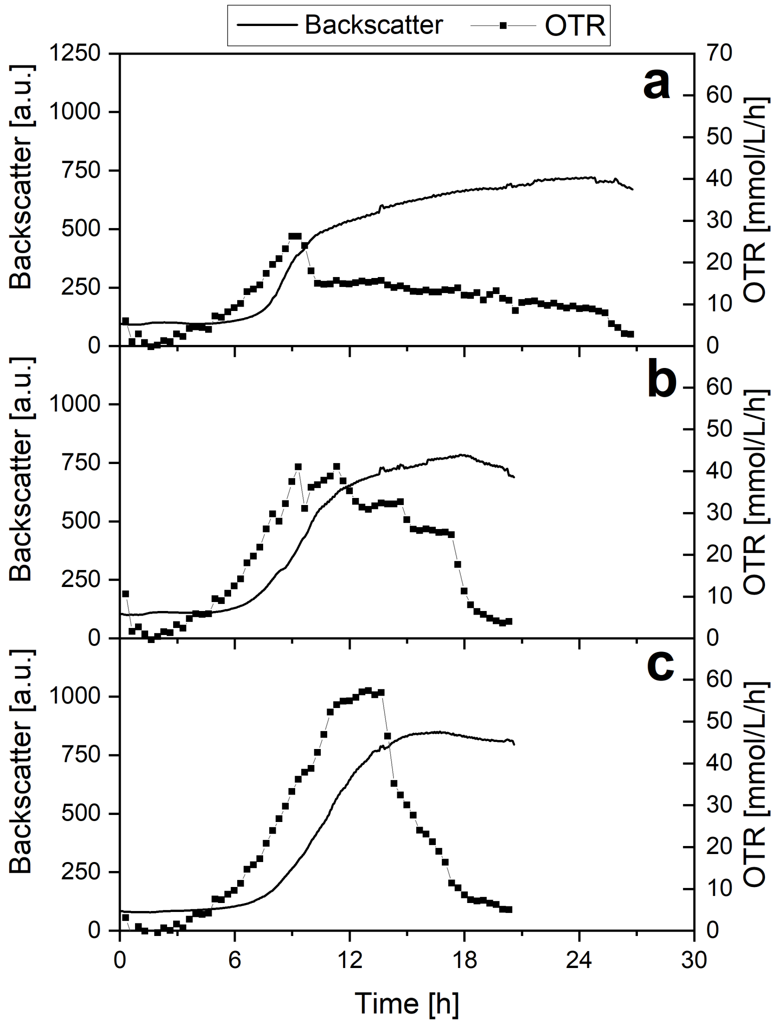

Figure S10. Repetition of the experiment illustrated in Figure 5: Oxygen transfer rate (OTR) and backscatter of shake flask cultivations of *S. pasteurii* in **(a)** chemically defined medium, **(b)** improved chemically defined medium and **(c)** improved YE medium. Average pH of all cultures: 9.32 ± 0.05. Culture conditions: 250 mL shake flasks, filling volume 10 mL, shaking frequency 300 rpm, shaking diameter 50 mm and temperature 30 °C.

Supplementary file 11 – OTR and backscatter data from *S. pasteurii* cultivation in YE medium

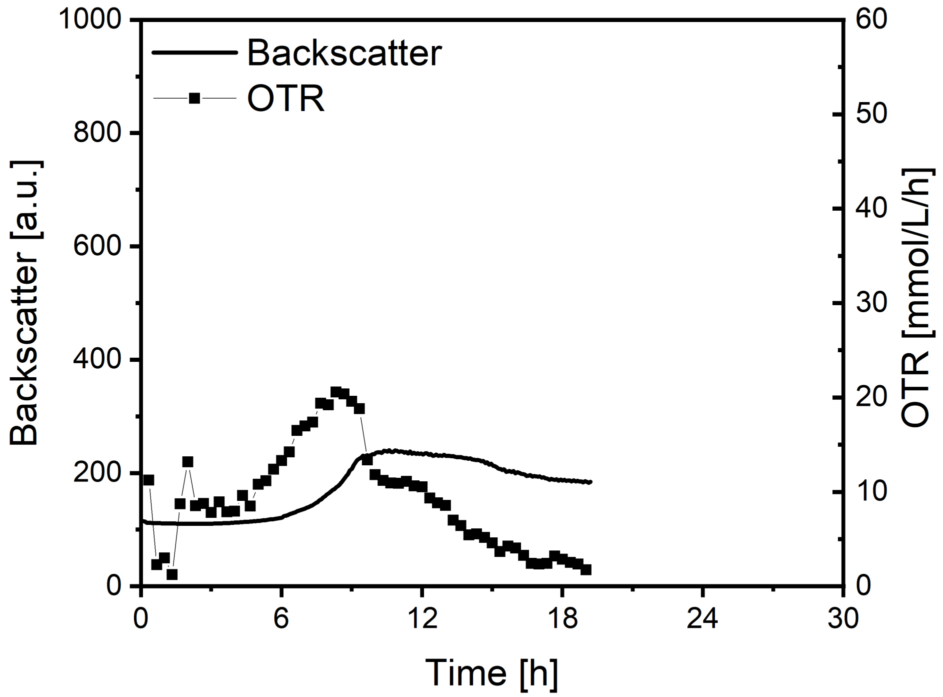

Figure S11. Oxygen transfer rate (OTR) and backscatter of shake flask cultivation of *S. pasteurii* in YE medium, cultivated parallel to the cultures illustrated in Figure 5. Culture conditions: 250 mL shake flasks, filling volume 10 mL, shaking frequency 300 rpm, shaking diameter 50 mm and temperature 30 °C.

Supplementary file 12 – Alterations made to the chemically defined medium

Table S12. Alterations made to the chemically defined medium by Müller *et al.*[27].

Component	Status	Reason for supplementation/omission
Urea	+	Urea is crucial for *S. pasteurii* growth.
Nickel chloride	+	Nickel is co-factor of urease. Addition of nickel has been proved beneficial for growth*.
Sodium chloride	+	NaCl was added as sodium source and for the osmotic pressure.
EDTA	+	EDTA was added as it improved solubility of the trace element solution.
L-glutamine	+	*S. pasteurii* is described to be auxotrophic for L-glutamine[30].
MOPS	-	MOPS was omitted as the strong buffer effect seemed to slow down bacterial growth. Due to ureolysis, pH increases rapidly after inoculation to approximately 9.3. This alkaline environment created by the alkaliphilic microorganism itself seems to be ideal for ATP generation[10].
Calcium chloride	-	Even low $CaCl_2$ concentrations led to $CaCO_3$ precipitation during cultivation, inhibiting bacterial growth.
Ascorbic acid	-	In preliminary experiments, ascorbic acid seem to inhibit growth. Zhang *et al.* made the same observations for *Helicobacter pylori*, also an ureolytic microorganism**.

(+) supplemented (-) omitted
Nucleobases/nucleosides and some vitamins were not available and therefore left out.
*) Onal Okyay, T. & Frigi Rodrigues, D. Optimized carbonate micro-particle production by sporosarcina pasteurii using response surface methodology. Ecol. Eng. 62, 168–174, DOI: 10.1016/j.ecoleng.2013.10.024 (2014).
**) Zhang, H.-M., Wakisaka, N., Maeda, O. & Yamamoto, T. Vitamin c inhibits the growth of a bacterial risk factor for gastric carcinoma:helicobacter pylori. Cancer 80, 1897–1903, DOI: https://doi.org/10.1002/(SICI)1097-0142(19971115)80:10<1897::AID-CNCR4>3.0.CO;2-L (1997).

Supplementary file 13 – Correlation between CDW and OD600

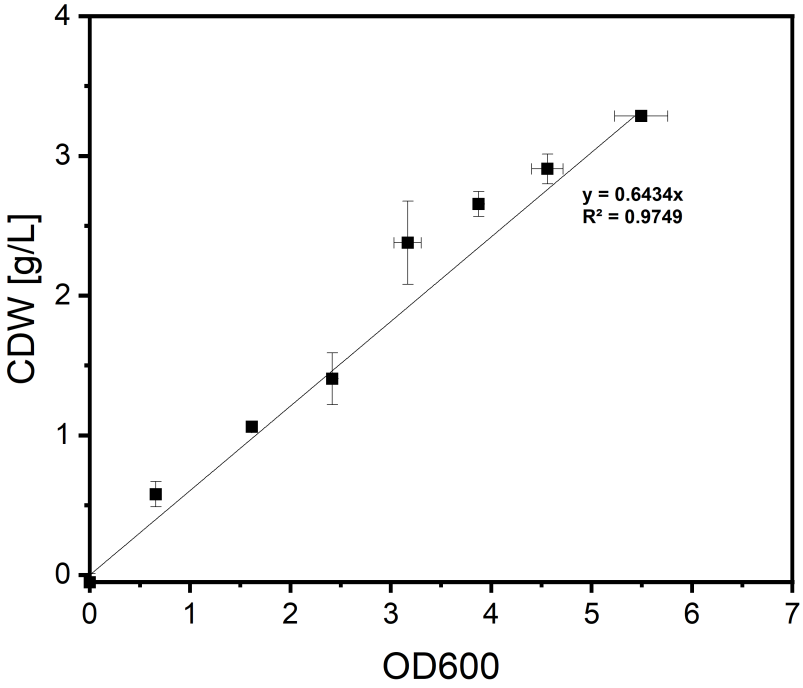

Figure S13. Correlation between CDW and OD600 of the biomass calibration obtained by a procedure similarly described by Kensy *et al.*[31]. *S. pasteurii* DSM33 was cultivated overnight in a 1 L shake flask, filling volume 250 mL, shaking frequency 300 rpm, shaking diameter 50 mm and temperature 30 °C in CaSo medium supplemented with glucose, K_2HPO_4, iron stock solution and micronutrient stock solution according to the concentrations described in Table 1. From this culture, a dilution series was pipetted. The OD600 from every dilution was determined with a photospectrometer (BioMate 3, Thermo Fisher Scientific, Waltham) as triplicates. Triplicates of the dilutions (15 mL) were washed twice with 9 % NaCl solution by centrifugation (6000 rpm, 10 min, Z300K centrifuge, Hermle, Wehingen). Cell pellets were then dried at 100 °C in open centrifuge tubes overnight. The samples were kept dry in a dessicator until weighing the tubes on an analytical balance (Sartorius R 300s, Göttlingen). The empty centrifuge tubes were weighed beforehand, allowing to calculate the pellet weight. With this, the CDW in g/L was calculated and correlated with OD600 data. Here, arithmetic mean values derived from triplicates (N = 3 for OD600, N = 3 for CDW) are shown. The error bars depict the standard deviation.

Supplementary file 14 – Urease activity standard curve

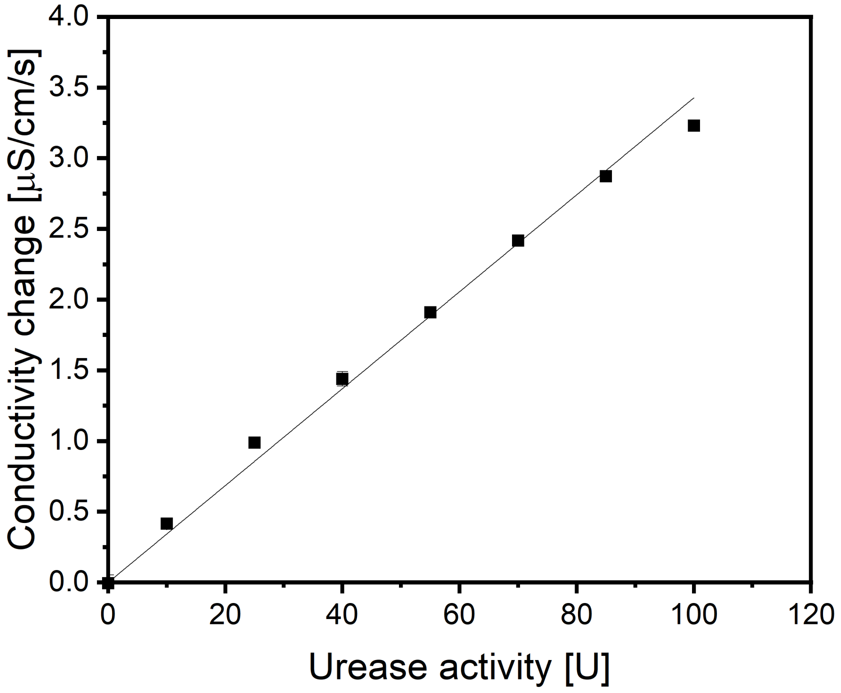

Figure S14. Urease activity standard curve of the conductivity assay derived from Whiffin[10]. The standard curve was generated using urease (Sigma aldrich, 666133-10KU) with known enzymatic activity. The manufacturer defines one unit as the amount of enzyme that will release 1 μmol of ammonia from urea per min at 25 °C, pH 7.0. Here, arithmetic mean values derived from technical replicates (N = 3) are shown. The error bars depict the standard deviation.

Three conductivity electrodes (InLab 751, Mettler Toledo, Giessen) were inserted in 22.5 mL of an 1.1 mol/L urea solution. After addition of 2.5 mL of *S. pasteurii* culture, measurement was started and carried out under constant stirring with a magnetic stirrer for 300 s. The average slope of linear conductivity increase was used to determine ureolytic activity. For the standard curve, 2.5 mL urease solution were used instead of the culture.
